# Structure-activity relationship of prevalent synthetic cannabinoid metabolites on hCB_1_ in vitro and in silico dynamics

**DOI:** 10.1038/s41401-025-01678-5

**Published:** 2025-11-03

**Authors:** Anna Åstrand, Emiliano Laudadio, Prince S. Gameli, Laura Martin, Jeremy Carlier, Francesco P. Busardò, Johan Dahlén, Xiongyu Wu, Peter Konradsson, Svante Vikingsson, Robert Kronstrand, Henrik Gréen

**Affiliations:** 1 Division of Clinical Chemistry and Pharmacology, Department of Biomedical and Clinical Sciences, Faculty of Medicine and Health Sciences, Linköping University, SE 581 85 Linköping, Sweden; 2 Department of Science and Engineering of Matter, Environment and Urban Planning, Polytechnic University of Marche, Ancona, Italy; 3 Department of Biomedical Sciences and Public Health, Polytechnic University of Marche, Ancona, Italy; 4 Institute of Chemistry and Bioanalytics, School of Life Sciences, University of Applied Sciences and Arts Northwestern Switzerland, CH 4132 Muttenz, Switzerland; 5 Department of Physics, Chemistry and Biology, Linköping University, SE 581 85 Linköping, Sweden; 6 Department of Forensic Genetics and Forensic Toxicology, National Board of Forensic Medicine, SE 587 58 Linköping, Sweden; 7 Center for Forensic Science Advancement and Application, RTI International, Research Triangle Park, NC 27709 USA; 8 Department of Biomedical and Clinical Sciences, Science for Life Laboratory, Linköping University, SE 581 85 Linköping, Sweden

**Keywords:** synthetic cannabinoid receptor agonists, active metabolites, pharmacodynamics, structure-activity relationship, in silico docking and molecular dynamics

## Abstract

Synthetic cannabinoids (SC) target the human cannabinoid receptor 1 (hCB_1_) and are extensively metabolized, but the metabolite activity on the hCB_1_ receptor after a SC intake is largely unknown. In this study we compared the in vitro hCB_1_ receptor activity of 26 metabolites of the synthetic cannabinoid receptor agonists (SCRA) JWH-018, AM-2201, THJ-018 and THJ-2201 as a model system for SC metabolite activity to elucidate their structure-activity relationships. The efficacy and potency of metabolites were assessed using an AequoScreen hCB_1_ receptor assay in triplicates and 7–8 concentration points (20 µg/mL–9.5 ng/mL) were used to construct dose-response curves and to determine EC_50_ and *E*_max_. In silico docking and molecular dynamics were performed using a model of the active form of the hCB_1_ receptor with all the metabolites. Final poses were simulated to assess stability under physiological conditions. We showed that carboxylic acid metabolites and 2-hydroxyindole biotransformational products were inactive, while 5-hydroxypentyl SCRA metabolites decreased efficacy to <70%, qualifying them as partial agonists. Eighteen metabolites retained >70% efficacy of their parent compound. Metabolite potencies ranged from 13–3500 nM where the most potent were the 4-hydroxypentyl derivatives of THJ-2201 and THJ-018 and the 4-hydroxyindole derivatives of AM-2201 and JWH-018, also known to be prevalent in vivo metabolites. The efficacy data from in silico experiments were correlated with the in vitro results demonstrating a linear trend (*R*^2^ = 0.9457), significant (*P* < 0.0001) at the 95% confident interval between the binding energies and efficacies of the compounds investigated. In silico analysis with docking and molecular dynamics simulations showed that active metabolites maintained a minimum of six amino acid interactions involving all substructures. The in silico molecular dynamics simulations revealed that the efficacy and potency seemed to be driven by a complex network of hydrophobic weak amino acid-ligand interactions. Most prevalent were CH-π interactions and π-π stackings. This study demonstrates the clear structure-activity relationships well correlated to the molecular dynamics simulations, suggesting that metabolites, especially the 4-hydroxy pentyl metabolites, may contribute to the overall effect of SCs in vivo.

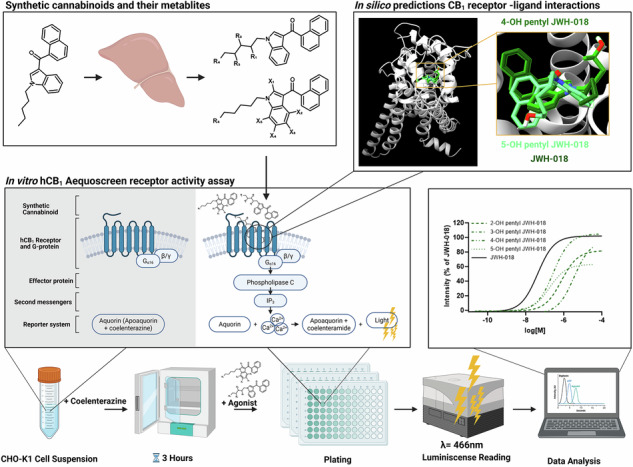

## Introduction

Synthetic cannabinoids (SC) were initially synthesized for research purposes with the aim of finding new pain relief medicine by selectively targeting human synthetic cannabinoid 2 (hCB_2_) receptors [1]. The Huffman laboratory researched a series of alkyl indoles (JWH series) for binding affinities on CB_1_ and CB_2_ to find a common pharmacophore [1]. Makriyannis and colleagues investigated among other factors, the effect of an incorporated fluorine at the terminal carbon of the alkyl indoles (AM series). However, the compounds were found to be non-selective, also activating CB_1_ receptors, showing psychoactive effect and thus having abuse potential [2].

**Fig. 1:**
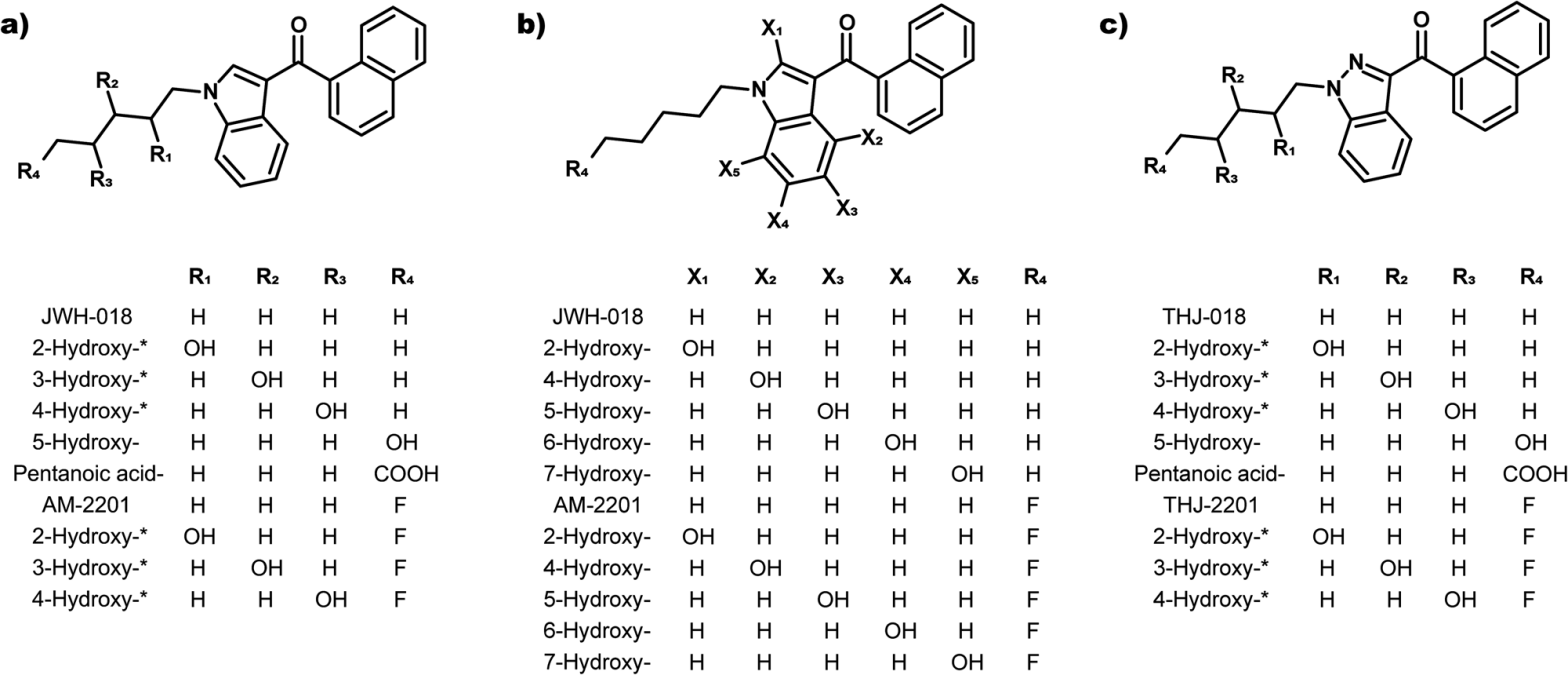
Structures of JWH-018, AM-2201, THJ-018, THJ-2201 and their metabolites. **a** Hydroxypentyl metabolites for indole SC, **b** Hydroxyindole metabolites, **c** Hydroxypentyl metabolites for indazole SC.

In 2008, JWH-018 (1-naphthalenyl(1-pentyl-1H-indol-3-yl)-methanone) was rediscovered as a drug of abuse when it was first identified as the active ingredient in Spice [3]. Since then, clandestine laboratories have produced a wide range of SC and at the end of 2023, the total number of cannabinoids monitored by European Union Drugs Agency (EUDA) were 254 [4]. The scheduling of JWH-018 paved way for similar SC. The most common structural elements of SC are indole or indazole cores with pentyl or 5-fluoropentyl tail groups [5]. Among the structural analogs of JWH-018 identified in forensic samples are the fluoro analog of JWH-018, AM-2210 ([1-(5-fluoropentyl)-1H-indol-3-yl]-1-naphthalenyl-methanone), the indazole analog THJ-018 (1-naphthalenyl(1-pentyl-1H-indazol-3-yl)-methanone) and the fluoro analog of THJ-018, THJ-2201 ([1-(5-fluoropentyl)-1H-indazol-3-yl]-1-naphthalenyl-methanone). These structurally related SC (Fig. 1) were first encountered by EUDA in 2011 (AM-2201), 2014 (THJ-018) and 2013 (THJ-2201) [6].

To date, JWH-018 is commonly used as reference, comparing its characteristics to novel SC in research literature and governmental reports [7–9]. The new role as reference increased the demand for more in-depth data to completely understand its pharmacology including metabolite hCB_1_ activity. Several research studies report JWH-018 metabolism, affinity and potency on the hCB_1_ receptor, mapping reported concentrations in authentic samples and adverse effects after intake [10–12]. Prevalent phase I metabolite pathways were monohydroxylation, resulting in positional isomers on the pentyl tail or on the indole core (for JWH-018 and AM-2201) as well as carboxylation on the pentyl tail (18, 23–25). These data enabled, e.g., synthesis of metabolite standards for forensic research [13–17].

Selected SC metabolites have been investigated for CB_1_ binding affinities and efficacy in vivo using a rat model and in vitro. 4- and 5-hydroxypentyl metabolites of JWH-018, JWH-122, JWH-210 and PB-22 were found to be active CB_1_ agonists, in contrast to their pentanoic acid metabolites or the carboxylic acid PB-22, which is inactive on the hCB_1_ receptor but activated the serotonin receptor 5-HT_2A_ [13, 18, 19]. 4-, 5-, 6- and 7-Hydroxyindole metabolites of JWH-018 were found to be active on the CB1 receptor [19]. Moreover, beta-D-glucuronide 5-hydroxypentyl JWH-018 has been reported as a neutral antagonist on hCB_1_ [20]. It is hypothesized that active SC metabolites are responsible for prolonging the effects of SC or are implicated in SC adverse reactions [21, 22].

Although some pharmacological data on SC and their metabolites exist, there is a lack of knowledge regarding the effect of metabolites in humans and on the hCB_1_ receptor. SC metabolites are often position isomers formed in varying abundances. As it is laborious to elucidate the exact structures of the metabolites from each SC, synthesize all metabolite standards and determine receptor activity, larger pharmacological studies on SC metabolites are scarce. However, such investigations would provide further knowledge on the pharmacological effects of existing SC, which is crucial for tracking SC in drug screens and for enabling the synthesis of relevant metabolite standards as reference materials.

In this study, we investigated and compared the potential activity of metabolites from JWH-018, AM-2201, THJ-018 and THJ-2201 on the hCB_1_ receptor. The parent compound and metabolite structures are shown in Fig. 1. By determining efficacy and potency of a large set of metabolites using a functional hCB_1_ receptor assay, we were able to investigate structure-activity relationships for the compounds. Moreover, we assessed the receptor-ligand interactions and binding characteristics in silico using docking and molecular dynamic simulations, with the aim of identifying the amino acids binding to the ligand and using the results to explain the differences observed in hCB_1_ activation induced by the metabolites.

## Materials and methods

### Drugs and chemicals

JWH-018, THJ-018, AM-2201, THJ-2201, 4- and 5-hydroxypentyl JWH-018, 4-hydroxypentyl AM-220, pentanoic acid JWH-018, 2-, 5-, 6- and 7-hydroxyindole AM-2201, 2-, 5-, 6- and 7-hydroxyindole JWH-018 were purchased from Cayman Chemicals (Ann Arbor, MI, USA). All other metabolite standards (presented in Fig. 1) were synthesized at Linköping University and their synthetic schemes and proof of purity in the form of NMR spectra, UV-Vis chromatogram and mass spectra are shown in Supplementary Material A. Stock solutions at 1 mg/mL were prepared in acetonitrile or methanol and stored at −20 °C. Transparent Dulbecco’s modified Eagle’s medium/Ham’s F-12 Nutrient Mixture (DMEM/Ham’s F12) supplemented with 15 mM HEPES and *L*-glutamine were purchased from Thermo Fisher (Gothenburg, Sweden). Trypsin was also purchased from Thermo Fisher Scientific (Gothenburg, Sweden). Adenosine triphosphate (ATP), digitonin, fetal bovine serum (FBS) and protease-free bovine serum albumin (BSA) were supplied by Fluka (Sigma-Aldrich, Stockholm, Sweden). Coelenterazine was from Nanolight Tech (Pinetop, AZ, USA). Stock solutions of 500 μM coelenterazine were prepared in methanol (and protected from light), 50 mM digitonin in DMSO and 10 mM ATP in Milli-Q water were stored at −20 °C.

### Cell lines and receptor assay

Calcium sensitive AequoScreen recombinant CHO-K1 cells expressing the hCB_1_ receptor were purchased from Perkin Elmer (Waltham, MA, USA) and stored in liquid nitrogen prior to culturing. The cell line was in culture for less than two months before the analysis was performed. The receptor assay was carried out according to the manufacturer’s recommendations. Specifically, AequoScreen recombinant CHO-K1 cells were transferred in DMEM/Ham’s F12 with 0.1% BSA. The cell suspension was spun down and the supernatant was discarded. After resuspension with 1 mL DMEM/Ham’s F12 medium, cells were diluted in assay medium to a final concentration of 3 × 10^5^ cells/mL. Coelenterazine, final concentration 2.5 µM, was added to the cell suspension and the cells were incubated for 3 h (dark, room temperature) prior to flash luminescence analysis (reading time 25 s/well) using a Spark 10 M with injector (Tecan, Switzerland).

### In vitro efficacy and potency determination of drugs and metabolites

To determine efficacy and potency of each drug and metabolite, hCB_1_ receptor activity was measured in triplicates at 8 concentrations ranging from 20 µg/mL to 76 ng/mL or 9.5 ng/mL. JWH-018 at 20 µg/mL (triplicate) was used as control and for normalizing data within plates. Negative (assay media) and positive controls (10 mM ATP and 100 mM digitonin) were analyzed in quadruplicate. A full dose-response curve of JWH-018 was analyzed at least every second day of experiments and was used to compare data obtained from different days. Each substance was analyzed on three different occasions (unless otherwise stated in Table 1) with freshly made dilutions.

**Table 1:** Drug or metabolite efficacy expressed as % of JWH-018 activity with 95% confidence interval and EC_50_ with 95% confidence interval of the drugs and metabolites are presented along the results (*P*-values) from one-way ANOVA at 95% significance level with Dunnet’s multiple comparisons* post hoc* test.

Compound name	Efficacy (% JWH-018 activity)	95% C.I.			*P*-values Efficacy	EC_50_ (nM)	95% C.I.			*P*-values EC_50_
AM-2201	99.67	96.23	–	103.10	reference	36.43	27.94	–	47.75	reference
2-OH pentyl AM-2201^a^	95.08	93.46	–	96.72	0.3321	375.30	337.20	–	417.80	<0.0001
3-OH pentyl AM-2201	100.30	98.06	–	102.50	0.9925	220.80	192.80	–	252.90	<0.0001
4-OH pentyl AM-2201	97.08	95.28	–	98.88	0.5072	63.65	56.25	–	72.03	0.0205
2-OH indole AM-2201	0.26	−0.57	–	1.23	<0.0001	–	–	–	–	–
4-OH indole AM-2201^c^	94.57	90.76	–	98.42	0.1581	43.70	32.62	–	58.45	0.682
5-OH indole AM-2201	93.44	89.63	–	97.31	0.0923	306.50	239.00	–	393.40	<0.0001
6-OH indole AM-2201	94.04	91.88	–	96.22	0.1399	122.20	105.50	–	141.80	0.0002
7-OH indole AM-2201	109.80	105.70	–	113.90	0.0053	282.20	227.10	–	350.40	<0.0001
JWH-018^d^	102.00	100.80	–	103.20	reference	42.41	39.09	–	46.03	reference
2-OH pentyl JWH-018^b^	82.05	76.82	–	87.46	<0.0001	749.80	526.60	–	1070.00	<0.0001
3-OH pentyl JWH-018	95.95	91.15	–	101.00	0.0107	3506.00	2881.00	–	4255.00	<0.0001
4-OH pentyl JWH-018	104.90	101.70	–	108.20	0.3332	288.30	235.50	–	354.00	<0.0001
5-OH pentyl JWH-018	63.06	60.15	–	66.05	<0.0001	177.90	129.90	–	245.40	<0.0001
Pentanoic acid JWH-018^b^	0.23	–		–	–	–	–		–	–
2-OH indole JWH-018	−0.38	−0.76	–	–	<0.0001	–	–	–	–	–
4-OH indole JWH-018	89.78	87.19	–	92.39	<0.0001	83.58	69.61	–	100.40	0.0143
5-OH indole JWH-018	88.92	84.70	–	93.32	<0.0001	1132.00	889.70	–	1446.00	<0.0001
6-OH indole JWH-018	59.31	55.92	–	62.78	<0.0001	459.90	345.70	–	608.70	<0.0001
7-OH indole JWH-018	15.81	13.46	–	18.25	<0.0001	–	–	–	–	<0.0001
THJ-018	96.34	90.64	–	102.30	reference	48.80	30.56	–	77.62	reference
2-OH pentyl THJ-018^b^	93.64	91.32	–	95.99	0.7021	1108.00	980.60	–	1252.00	<0.0001
3-OH pentyl THJ-018	99.82	98.00	–	101.60	0.4553	202.10	180.90	–	225.80	<0.0001
4-OH pentyl THJ-018	98.47	96.58	–	100.40	0.8011	174.90	156.10	–	196.00	0.0002
5-OH pentyl THJ-018	67.29	64.25	–	70.39	<0.0001	274.10	208.90	—	358.60	<0.0001
Pentanoic acid THJ-018^b^	11.68	–		–	–	–	–		–	–
THJ-2201	92.83	90.51	—	95.17	reference	30.65	25.81	–	36.39	reference
2-OH-pentyl THJ-2201	86.95	84.53	—	89.39	0.0144	402.60	343.50	–	471.40	<0.0001
3-OH-pentyl THJ-2201^c^	100.60	98.55	—	102.70	0.006	158.90	139.50	–	181.00	<0.0001
4-OH-pentyl THJ-2201	99.21	97.56	—	100.90	0.0096	13.71	12.01	–	15.67	0.0005

Efficacy and potency of each metabolite were compared to their respective drug, marked as reference in the table.

^a^1 ×  3 replicates.

^b^2 ×  3 replicates.

^c^4 × 3 replicates.

^d^13 × 3 replicates.

Potency was determined from dose-response curves by calculating EC_50_ with 95% confidence interval. *E*_max_ was used to determine efficacy.

### In silico ligand-hCB_1_ docking

The structure of each metabolite and drug was generated and minimized using UCSF Chimera [23]. The three-dimensional structure of the hCB_1_ receptor was obtained from the 6N4B pdb file [24] and modified to include the N-domain by using I-Tasser [25]. The generated files were then used to perform molecular docking.

The ligand-hCB_1_ interactions were investigated to rationalize the binding mode of the ligands using AutoDock Suite 4.2 [26]. Autodock tools were used to add polar hydrogen atoms and partial charges to the receptor and ligands [27]. Atomic solvation parameters and fragmental volumes for the hCB_1_ were assigned using the Addsol tool, included in the program package. Flexible torsions of the ligands were allocated with the Autotors module and all dihedral angles were allowed to rotate freely. Affinity grid fields were generated using the auxiliary program Autogrid. A grid field of 50 Å × 48 Å × 48 Å and the resulting docked conformations were clustered into families of similar binding modes, with a root mean square deviation (RMSD) clustering tolerance of 2 Å. The most populated docking conformations with more negative binding energy were considered the most stable orientations of each compound in the hCB_1_ receptor pocket. The above-mentioned binding energy represents the sum of the intermolecular contributions and the internal energy, in terms of the intermolecular and the torsional energetic values [28]. The binding energies of docked complexes were calculated by an empirical free energy force field with a Lamarckian genetic algorithm (LGA), which provides a fast prediction of conformation and free energy. This calculated free binding energy is related to the inhibition constant (*K*_i_) through the thermodynamic law ∆*G* = −RT ln *K*_i_.

### Molecular dynamics modeling

Molecular dynamic (MD) simulations were conducted for 7-hydroxyindole AM-2201, pentanoic acid JWH-018 and 7-hydroxyindole JWH-018 with high (87%), medium (57%) and low (43%) population percentages, respectively, to preliminarily assess whether MD simulations were needed for all ligand-hCB_1_ complexes. As a result, all compounds with population percentages less than 74% were simulated through MD to achieve a dynamically stable pose. Details of all validity assessments performed in this study can be found in Supplementary Material B. A simulation box of 6.82 nm × 6.82 nm × 8.50 nm was generated using Charmm-GUI [29] and periodic boundary conditions (PBCs) along all axes were utilized. A membrane composed of 1-palmitoyl-2-oleoyl-sn-glyceo-3-phosphocholine (POPC, 146 lipids) was modeled and hCB_1_ was inserted inside the lipid membrane using the correct coordinates obtained by Positioning of Proteins in Membranes (PPM) server [30]. The simulation box was solvated by 16,582 TIP3 H_2_O molecules [31] and 44 Na^+^ ions with 52 Cl^−^ counterions were included to reach the physiological condition of 0.15 M NaCl and to neutralize the net charge of hCB_1_.

For each ligand, a new model was generated, and a massive minimization step was performed followed by six cycles of equilibration. Thus, 200 ns of (MD) simulations were carried out for the production phase using a thermodynamic ensemble in which the number of molecules (N), the values of Pressure (P) and of the Temperature (T) were constant throughout the MD time.

For the equilibration step, a temperature of 298 K was applied whiles 310 K was employed during the 200 ns MD simulations. This approach, also called NPT ensemble, was used to treat each system in semi-isotropic conditions. All simulations were performed using GROMACS 2023.3 version and CHARMM36 force field [32]. Finally, the trajectories were analyzed using Visual Molecular Dynamics (VMD) [33] and UCS Chimera software. The population percentages for the binding poses of the ligands were all above 76% when the ligand-hCB_1_ complexes were analyzed for number and type of interactions [23]. The interaction pattern of each ligand was identified by considering all residues within 3 Å of the compound position, analyzing in detail the nature of the interactions based on the surfaces of the functional groups in contact. In this study, no intramolecular interactions were investigated. The details of the in silico study is presented in Supplementary Material B.

### Statistical analysis

For data analysis, Excel by Microsoft version 16.24 and GraphPad Prism for Windows 64-bit version 8.1.1 (330) were used. The raw data from the luminescence assay were transferred into Excel, where signals from each well were summed up, blank signals subtracted and normalized to the reference compound JWH-018. Next, the concentration of the compounds was converted to molar and divided into sets according to when the experiments were performed. All data was transferred to GraphPad Prism where dose-response curves of JWH-018 were drawn and EC_50_ calculated for JWH-018 separately for all sets. Efficacy and EC_50_ values were normalized to JWH-018 to compensate for between-run variability, allowing comparisons across the whole data set. Parent drug and metabolites showing less than 20% efficacy were regarded as inactive and excluded from further testing. Efficacy and potency were statistically compared using One-Way ANOVA with multiple comparisons at the significance level of *P* = 0.05. The EC_50_ values of the metabolites were compared to the parent drugs using Dunnett’s multiple comparison test. Metabolite EC_50_ values were groupwise tested using Tukey’s multiple comparison test.

## Results

### Efficacy of AM-2201, JWH-018, THJ-018, THJ-2201 and their metabolites

**Fig. 2:**
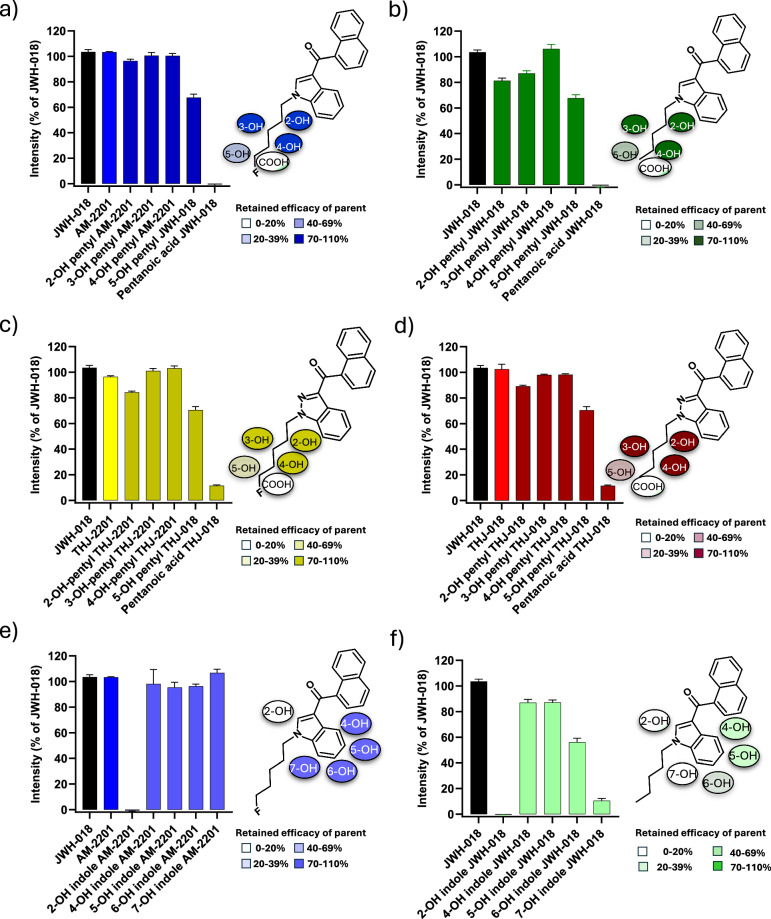
Metabolite efficacy expressed as % of the activity of JWH-018 at the concentration of 20 µg/mL. Error bars represent standard error of the mean (SEM). Hydroxypentyl and hydroxyindole metabolites are grouped according to their parent and hydroxylated substructure. **a** AM-2201 and monohydroxylated pentyl metabolites, **b** JWH-018 and monohydroxylated pentyl metabolites, **c** THJ-018 and monohydroxylated pentyl metabolites and **d** THJ-2201 and monohydroxylated pentyl metabolites. **e** AM-2201 and monohydroxylated indole metabolites. **f** JWH-018 and monohydroxylated indole metabolites. 5-hydroxypentyl JWH-018 and pentanoic acid JWH-018 are depicted in both (**a**, **b**) because they can originate from both JWH-018 and AM-2201. Likewise, 5-hydroxypentyl THJ-018 and pentanoic acid THJ-018 are shown both in (**c**, **d**). The chemical structures of the parent with all possible hydroxy positions are depicted.

**Fig. 3:**
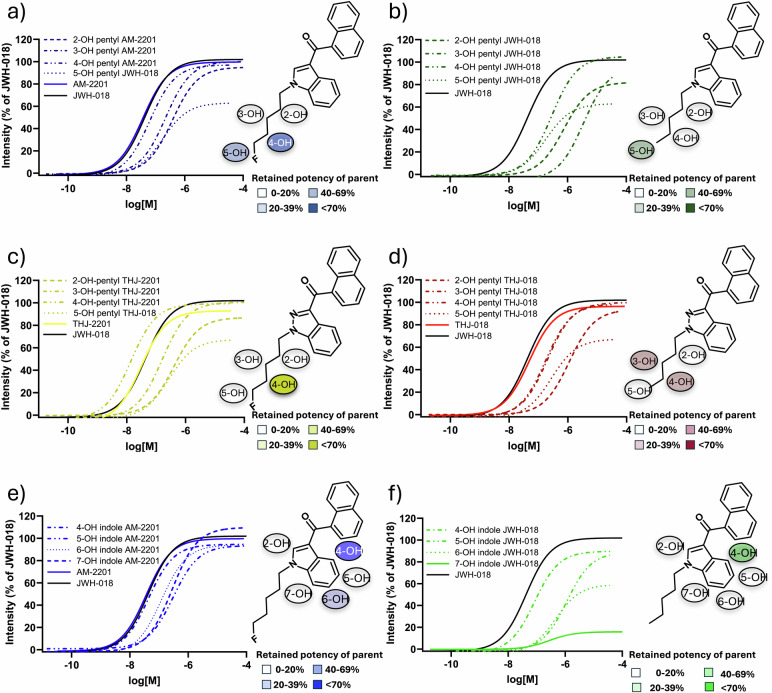
Dose-response curves for the parent and metabolites with JWH-018 present as reference are depicted and grouped according to their parent and hydroxylated substructure. **a** AM-2201 and monohydroxylated pentyl metabolites, **b** JWH-018 and monohydroxylated pentyl metabolites, **c** THJ-018 and monohydroxylated pentyl metabolites and **d** THJ-2201 and monohydroxylated pentyl metabolites. **e** AM-2201 and monohydroxylated indole metabolites. **f** JWH-018 and monohydroxylated indole metabolites. The chemical structures of the parent with all possible hydroxy positions are depicted.

Data on efficacy (*E*_max_) are summarized in Table 1 and Fig. 2. For the active metabolites, dose-response curves used to determine the potency (EC_50_) on the hCB_1_ receptor are presented in Fig. 3. Calculated EC_50_ and *P*-values from statistical testing are presented in Table 1. Compared to the *E*_max_ of JWH-018 (102%, reference), the *E*_max_ of AM-2201 (99.67%, *P*-value 0.57) at 95% confidence level was not significantly different. In comparison, the *E*_max_ of THJ-018 (96.39%, *P*-value 0.03) and THJ-2201 (92.83%, *P*-value 0.0004) were significantly lower. The four drugs were found to have both hCB_1_ active pentyl- and indole-hydroxy metabolites with efficacies comparable to full agonists and their respective parent drugs. In total, 21/26 metabolites activated the hCB_1_ receptor.

All 2-, 3- and 4-hydroxypentyl metabolites retained hCB_1_ activity with *E*_max_ ranging from 82% to 105% of their respective parent SC (Fig. 2a–d and Table 1). In contrast, both 5-hydroxypentyl metabolites had reduced *E*_max_ (63% and 67%) and both pentanoic acid metabolites were inactive (*E*_max_ < 20%). Among the hydroxyindole metabolites (Fig. 2e, f), 4-hydroxy- and 5-hydroxyindole metabolites retained hCB_1_ activity with *E*_max_ 89%–95% of JWH-018 and AM-2201. On the contrary, both 2-hydroxyindole metabolites were inactive (*E*_max_ < 20%). Of interest, the 6- (*E*_max_ 59%) and 7-hydroxyindole metabolites (*E*_max_ < 20%) of JWH-018, showed less efficacy than those of AM-2201 (*E*_max_ 94% and 110%, respectively).

### Potency of AM-2201, JWH-018, THJ-018, THJ-2201 and their metabolites

All four SC showed similar potency with EC_50_ ranging from 30.7 nM (THJ-2201) to 48.8 nM (THJ-018). The differences were not significant, except for THJ-018 and THJ-2201 (*P* value 0.048) (Table 1). Hydroxypentyl metabolites had significantly lower potency than their parent SC with EC_50_ 3.6 to 83 times higher (Table 1). The exception was the fluorinated 4-hydroxypentyl metabolites of AM-2201 and THJ-2201. While less potent (*P* value 0.02), the ECs_50_ value of 4-hydroxypentyl AM-2201 was only 1.7 times higher than that of AM-2201. Even more pronounced, 4-hydroxypentyl THJ-2201 was the only metabolite more potent (2.2×, *P* value 0.0005) than the parent SC. All hydroxyindole metabolites were significantly less potent than their respective parent SC (JWH-018 or AM-2201), except 4-hydroxyindole AM-2201 which has similar potency as AM-2201 (44 and 36 nM, respectively, *P* value 0.682).

Further investigation of the potency of the active hydroxyindole metabolites showed few position-specific trends. However, the 4-hydroxyindole metabolites of both SC were the most potent (83% of AM-2201 and 51% of JWH-018, respectively). For AM-2201 6-, 7- and 5- hydroxyindole metabolites retained over 12% of the potency of the parent. 5- and 6- hydroxyindole JWH-018 dropped to 4% of the potency of JWH-018. The potencies of hydroxyindole metabolites of AM-2201 were in general more potent than their counterpart of JWH-018.

### Molecular docking and dynamic simulation of drug/metabolite-hCB_1_

**Fig. 4:**
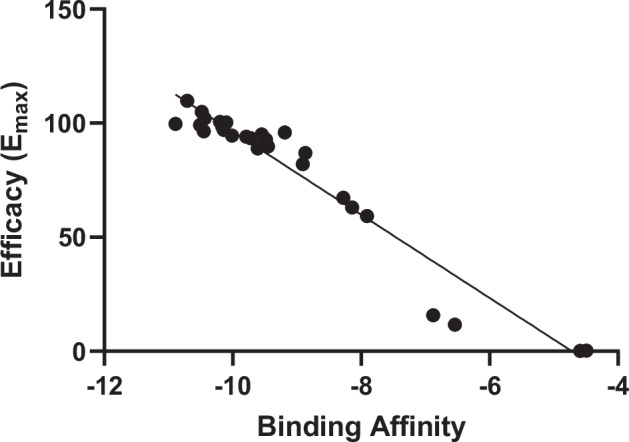
Correlation between efficacy (% of JWH-018) and binding affinities (kcal/mol). Calculated *R*^2^-value is included.

Receptor docking was simulated for all ligands (SC and metabolites); binding energy, population percentage and predicted *K*_i_ are presented in Table 2. For the metabolites with a chiral center (marked with * in Fig. 1), simulations were performed for both enantiomers separately; however, the racemates of the enantiomers were calculated and used for comparison with the in vitro data. Binding energies ranged from −10.89 kcal/mol for AM-2201 to −4.29 kcal/mol and the predicted *K*_i_ values were from 6.45 nM to 1599 nM. Only small differences in binding energy were observed for 2-, 3- and 4-hydroxypentyl metabolites (Table 2) and both the *S*- and *R*- forms generally exhibited similar affinities to the receptor. As seen in Fig. 4, a linear trend (*R*^2^ = 0.9457), significant (*P* < 0.0001) at the 95% confident interval, was found when efficacy data were plotted against the binding energies. The binding energy is a measure of binding affinity: the lower the binding energy, the higher the binding affinity for the compound binding to the receptor. A correlation was also found between the calculated *K*_i_ values and the in vitro measured log EC_50_ (*R*^2^ = 0.2656) significant (*P* < 0.0084) at the 95% confident interval (Supplementary Material B).

**Table 2:** Binding Energy, Population Percentage and predicted *K*_i_ of each compound.

Name	Binding Energy (kcal/mol)	% Population	Predicted *K*_i_ (nM)
AM-2201	−10.89	91	10.42
2-OH pentyl AM-2201	−10.05/−9.05	84 / 68	43.16/109.04
3-OH pentyl AM-2201^†^	−9.91/−10.29	84 / 88	55.09/25.01
4-OH pentyl AM-2201^†^	−9.99/−10.28	86 / 81	60.67/26.91
2-OH indole AM-2201^†^	−4.49	89	1461.45
4-OH indole AM-2201^†^	−10.01	83	49.22
5-OH indole AM-2201	−9.72	89	71.23
6-OH indole AM-2201	−9.79	83	67.17
7-OH indole AM-2201	−10.71	87	6.45
JWH-018	−10.44	90	22.27
2-OH pentyl JWH-018 ^††^	−9.26/−8.56	90/85	93.42/128.91
3-OH pentyl JWH-018 ^††^	−9.36/−9.01	93/87	109.68/139.02
4-OH pentyl JWH-018 ^††^	−10.51/−10.45	93/91	17.50/20.56
5-OH pentyl JWH-018 ^†^	−8.14	90	142.17
Pentanoic acid JWH-018	−4.59	87	1371.86
2-OH indole JWH-018 ^†^	−4.29	82	1598.57
4-OH indole JWH-018	−9.45	87	119.21
5-OH indole JWH-018	−9.61	83	91.35
6-OH indole JWH-018 ^†^	−7.91	90	192.72
7-OH indole JWH-018 ^†^	−6.88	83	1109.88
THJ-018	−10.45	91	19.64
2-OH pentyl THJ-018^††^	−9.45/−9.59	82/84	118.89/91.76
3-OH pentyl THJ-018 ^†^	−10.09/−10.18	94/89	46.75/30.65
4-OH pentyl THJ-018 ^†^	−10.15/−10.16	87/80	36.18/35.12
5-OH pentyl THJ-018 ^†^	−8.28	87	138.34
Pentanoic acid THJ-018^†^	−6.54	83	1165.12
THJ-2201^†^	−9.48	83	88.95
2-OH pentyl THJ-2201	−9.25/−8.48	80/76	93.39/134.01
3-OH pentyl THJ-2201	−10.22/−10.18	89/84	31.9/30.64
4-OH pentyl THJ-2201	−10.47/−10.54	83/86	20.74/16.54

For the enantiomers, first *R*-, then *S*- configurations have been reported. Compounds highlighted with a dagger (^†^) are those for which the population percentage was low in initial docking and the reported binding energy, population density and predicted *K*_i_ values were refined with MD simulations. (^††^) indicates that both enantiomers were subjected to MD simulations.

### Identifying amino acids–ligand interactions

**Fig. 5:**
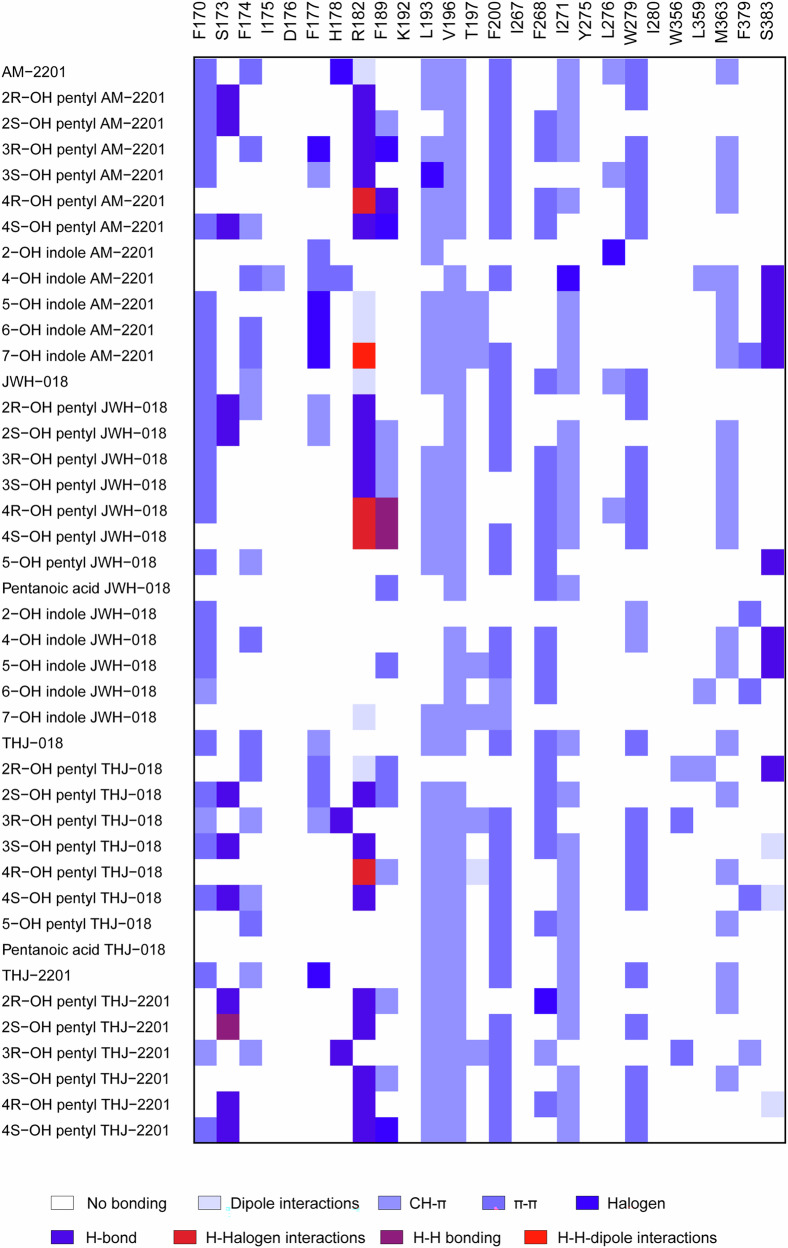
Summary of identified individual ligand-amino acid interactions, results from docking and molecular dynamics simulations. Amino acids present in the hCB_1_ active pocket were considered for possible interactions (x-axis). Each drug and metabolite are presented along the y-axis. Each color represents one of these types of interactions: H-halogen-dipole, H-halogen, H-H bond, H-bond, halogen, H/CH- π, CH- π, dipole-dipole interactions or π-π stacking.

Analyzing the binding mode of each compound allowed the identification of amino acids involved in the interaction, within the binding pocket of the hCB_1_ receptor. Figure 5 depicts an overview of all interactions between amino acids of the hCB_1_ receptor and the ligands following molecular docking and dynamic simulations. Predominant interactions were observed for the hydrophobic amino acids, such as Val196, Leu193, Met363 and Iso271 via CH-π interactions, while minor differences were observed between *R*- and *S*-enantiomers of the hydroxylated pentyl metabolites (Fig. 5).

**Fig. 6:**
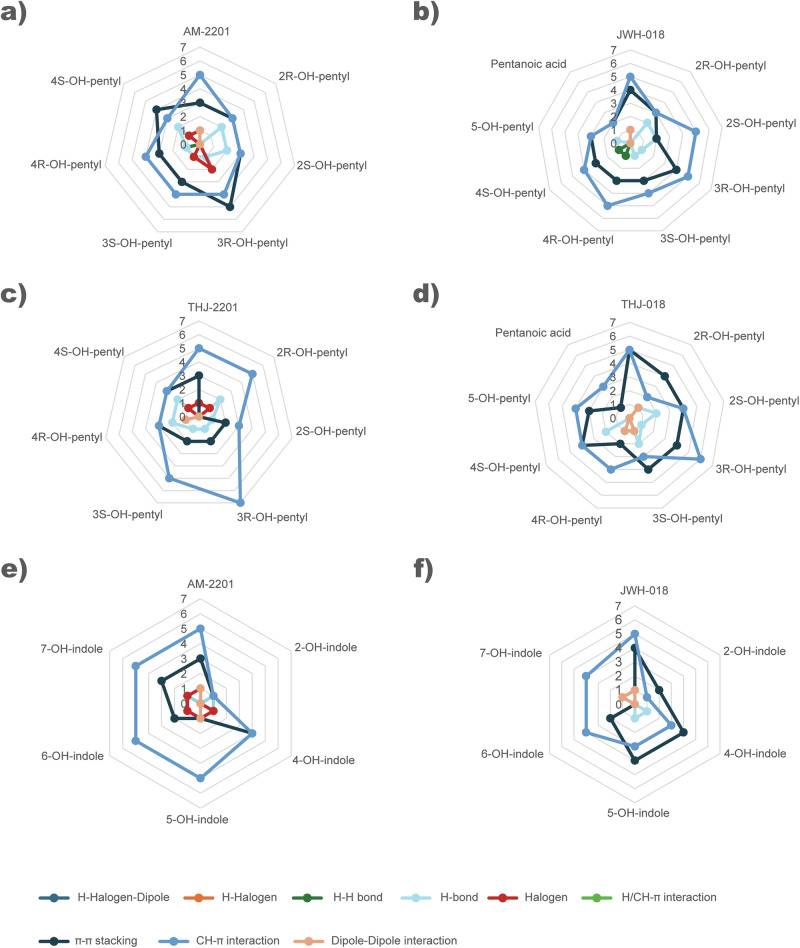
Summary of number and type of ligand-amino acid interactions grouped according to type of metabolite from each of the synthetic cannabinoids JWH-018, AM-2201, THJ-018 or THJ-2201. The represented types of interactions are H-halogen-dipole, H-halogen, H-H bond, H-bond, halogen, H/CH- π, CH- π, dipole-dipole interactions or π-π stacking. **a** AM-2201 and monohydroxylated pentyl metabolites, **b** JWH-018 and monohydroxylated pentyl metabolites, **c** THJ-2201 and monohydroxylated pentyl metabolites, **d** THJ-018 and monohydroxylated pentyl metabolites. **e** AM-2201 and monohydroxylated indole metabolites. **f** JWH-018 and monohydroxylated indole metabolites.

The total number and type of interactions between the hCB_1_ and ligands are depicted in Fig. 6. Arg182 had several interaction types with the parent drug and metabolites within the hCB_1_ binding pocket. Polar induced dipole-dipole interactions were found between Arg182 and AM-2201 and JWH-018, as well as their indole metabolites (5- and 6-OH indole AM-2201 and 7-OH indole JWH-018). However, 7-hydroxyindole AM-2201 had H-halogen-dipole interactions, a much stronger interaction, with this hydrophilic amino acid. H-bonds and H-halogen interactions with Arg182 were also identified for hydroxy-pentyl metabolites, except for 2*R*-OH-pentyl THJ-018, which showed a dipole-dipole interaction. Other interactions identified for the metabolites included H-H bonding and halogen interactions. For the parent drugs, halogen interaction involving the fluorine atoms of AM-2201 and THJ-2201 were with His178 and Phe177, respectively.

**Fig. 7:**
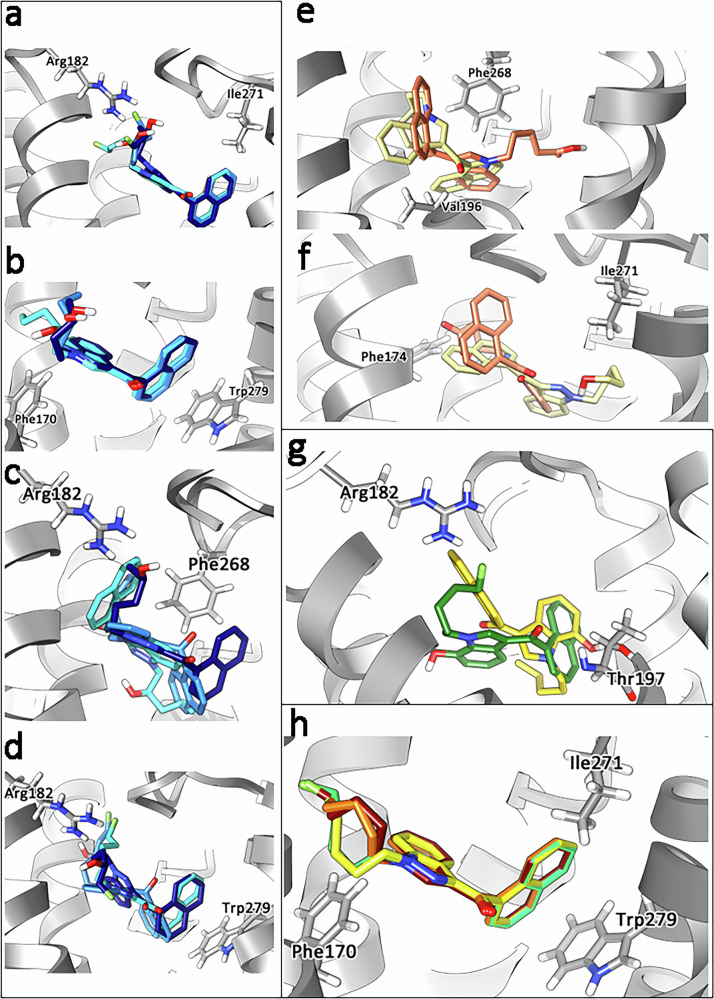
Visualization from docking experiment. Ligand configurations superimposed together with hCB_1_ receptor with selected amino-acid residues. Gradual rotation in configuration for the *R*-form of 2-, 3- and 4-hydroxy pentyl metabolites of AM-2201 (**a**), JWH-018 (**b**), THJ-018 (**c**), and THJ-2201 (**d**). For each panel, 2-, 3-, and 4- hydroxy pentyl are reported in cyan, dark blue and navy blue respectively. **e**, **f** Comparison of the configurations of 5-hydroxy pentyl JWH-018 (khaki) and pentanoic acid JWH-018 (coral). **g** Configurations of 7-hydroxy indole AM-2201 (light brown) and 7-hydroxy indole JWH-018 (green). **h** AM-2201 (dark red), JWH-018 (orange) THJ-018 (yellow) and THJ-2201 (green) superimposed in the active pocket of hCB_1_.

Compounds with low binding energy from the molecular docking approach reached ten or eleven explicit interactions with amino acids. In contrast, metabolites with higher kcal/mol showed no more than four amino acids interactions involved in the binding. At the end of the MD simulations, the number of amino acids involved was the same for each compound, even if accommodation in the binding site was always observed. All SC and their metabolites interacted with Val196 and Leu193 via CH-π interactions, with minor exceptions for enantiomeric pairs. Ser383 was found to interact with the core of active hydroxylated indole/indazole metabolites, except for 6-hydroxyindole JWH-018. Interestingly, this interaction was not observed for the parent compounds or pentyl metabolites. The amino acid patterns varied for sets of drugs/metabolites due to the presence of different functional groups. However, the molecules with related experimental efficacy exhibited similar interactions from our in silico docking and MD simulation study. The 2-, 3-, and 4-hydroxypentyl shared the overall configuration in the receptor but differed in amino acid interactions, which caused a slight rotation in comparison to one another (Fig. 7).

## Discussion

In this study, JWH-018, AM-2201, THJ-018 and THJ-2201 and their metabolites were screened for hCB_1_ efficacy and potency using a cell-based flash luminescence assay. This hCB_1_ receptor assay has previously been used to report on SC effects for both research purposes and to provide governmental agencies timely data aimed at speeding up the scheduling process of novel SC [8, 9, 18, 34].

In silico ligand-hCB1 simulations gave further indications on the behavior of the parent drug and metabolites within the receptor pocket. The molecular docking approach successfully reproduced the active conformation of the human CB_1_ receptor (hCB_1_) bound to the agonist methyl N-(1-((4-fluorobenzyl)-1H-indazole-3-carbonyl)-3-methyl-*L*-valinate (KCA), a co-crystallized ligand in the CB_1_ receptor structure (PDB ID: 6N4B), following remodeling to include the N-terminal domain [24]. The hCB_1_ receptor was remodeled in this study to ensure that likely interaction(s) between SC/metabolites and the N-terminal were not missed. RMSD value following simulation of the remodeled hCB_1_ and KCA residue was less than 0.9 Å demonstrating structural congruence.

It was found that the binding energy on the active form of the hCB_1_ receptor inversely correlates with the in vitro efficacy (Fig. 4). This finding indicates that the results from these two independent methods overlap, making it possible to extrapolate the in vitro results based on the in silico results. A weaker correlation between *K*_i_ and EC_50_ values was also observed (Supplementary Material B) which further strengthens this hypothesis. Thus, the predicted poses of the ligand in the hCB_1_ receptor after MD simulations could be used to further explain the differences in vitro efficacy and potency for each group of metabolites by looking at the number and type of interactions between the ligand and individual amino acids of the receptor. The found correlations between the in silico model and the experimental data suggest that this approach could be used for in depth studies of hCB_1_ activity of other SC and their metabolites too.

### Structure-hCB_1_ activity relationship of JWH-018, AM-2201, THJ-018, THJ-2201

Comparing ligand configuration in the hCB_1_ receptor for the SC reveals only minor changes. All SC were full agonists with nanomolar-range potencies in vitro. Krishna et al., 2024, previously reported key interactions between JWH-018, and other derivatives from the JWH-018 family, and hCB1 using Schrödinger’s docking approach and molecular dynamics simulations. Similar results were obtained for JWH-018, THJ-018, AM-2201, and THJ-2201, depicting interactions with the amino acids, Phe170, Phe174, Phe200, and Trp279 are pivotal in SC stability in the hCB1 receptor pocket [35]. The largest difference observed in pairwise comparisons between AM-2201 and JWH-018 configurations was that AM-2201 had a more linear orientation in the hCB_1_ receptor pocket due to the fluorine atom at the 5th carbon of the pentyl chain. The addition of the electronegative fluorine on the tail interacts with His178, resulting in one additional amino acid interaction and perhaps anchoring AM-2201 in the pocket, contributing to the overall stability. This slight difference does not affect the configuration of the core and head, as the orientation of the substructures overlap. Similarly, the tail of THJ-2201 was more linear as compared to that of THJ-018. In general, the introduction of a fluorine atom stabilizes the molecule by enabling stronger binding to the receptor due to its high electronegativity and lipophilicity, thereby influencing the pharmacological effects of a drug [36].

Comparing the configuration of the two 5-fluoro-pentyl SC reveals that AM-2201 and THJ-2201 have overlapping orientation in the hCB_1_ pocket. The difference in their binding energy likely stems from the presence of two additional amino acid interactions for AM-2201 and better stabilization of the binding by the indole core of AM-2201 compared to the indazole core of THJ-2201 (Fig. 7). Additionally, Yano et al., 2023, demonstrated the interaction with His178 may not only stabilize 5-fluoro-pentyl SCRA derivatives in the hCB1 pocket, but also may be important in activating the CB1 receptor, in essence acting as an on/off switch for 5-fluoro-pentyl SC analogues [37]. All in all, chemical modifications more important than those above-mentioned need to be introduced for the SC to give a different effect in vitro.

### Structure-activity comparison of hydroxypentyl metabolites

The length, bulkiness and constitution of the SC tail is found to be one of the key features to affect potency as well as efficacy [34]. 2-, 3- and 4-hydroxypentyl metabolites from all four SC in our study retained over 82.5% of the parent efficacy (Fig. 2). Moreover, we found that the addition of a hydroxyl group to the SC pentyl tail significantly reduces hCB_1_ potency in comparison to the parent. The exception seems to be the fluorinated 4-hydroxypentyl metabolites of AM-2201 and THJ-2201: for 4-hydroxypentyl AM-2201, the reduction in potency is minimal (1.7×), while for 4-hydroxypentyl THJ-2201 the potency increased (2.2×). Ranking the hydroxypentyl metabolites in order of potency reveals a different pattern (4-, 3-, 2-hydroxypentyl for AM-2201; 4-, 5-, 2-, 3-hydroxypentyl for JWH-018; 4-, 3-, 5-, 2-hydroxypentyl for THJ-018 and 4-, 3-, 2-hydroxypentyl for THJ-2201) compared to ranking them according to their efficacy or binding affinity.

Other in vitro studies previously reported JWH-018 as an agonist at nanomolar concentrations, and 4- and 5-hydroxypentyl JWH-018 as active metabolites, with 4-hydroxypentyl JWH-018 as the more efficacious and potent of the two. Pentanoic acid JWH-018 was found to be inactive in the same study [13]. These findings are consistent with the current study.

From our in silico results, there was not a clear amino acid interaction elucidating the underlaying mechanism of SC and their metabolites’ potency at the hCB_1_, although it is likely that multiplying interactions contributes to the stability of the compound within the receptor pocket, which in turn influences the compound’s potency based on the predicted *K*_i_ values.

A detailed analysis of the hCB_1_ predicted configurations for the hydroxypentyl metabolites reveals a trend attributable to the gradual shift (Fig. 7) of 2-, 3- and 4-hydroxypentyl metabolites in the hCB_1_ pocket. Interestingly, there was no noticeable difference between the *R*- and *S*- enantiomers of the metabolites. Regardless of this configuration, the metabolites in this study explicitly interacted with at least 8 amino acid in the hCB_1_ receptor pocket to be efficacious.

In contrast, both 5-hydroxypentyl metabolites caused a marked reduction in hCB_1_ efficacy and acted as a partial agonists, while the pentanoic acid metabolites were found to be inactive. In silico, the 5-hydroxypentyl and pentanoic acid metabolites of JWH-018 were oriented differently in comparison to JWH-018, as illustrated in Fig. 7, resulting in a reduced number of total interactions and a changed amino acid interaction pattern (Figs. 5, 6). Our results demonstrated that hydroxylation and carboxylation on the terminal carbon of the tail affect the efficacy, matching previously reported results from affinity [38] and efficacy experiments [13, 19, 39]. Notably, not all modifications on the fifth carbon of the tail seem to reduce efficacy. 5F-pentyl is one of the most common substructures for efficacious and potent SC and as discussed above, the pentyl and corresponding 5F-pentyl SC showed similar potencies in our study. The difference in observed efficacies could be due to the size, electronegativity and 3D structure of the different atoms or molecular groups. A hydroxyl group is larger than a fluorine atom despite having the same number of electrons. This is due to the high electronegativity of fluorine which keeps the electron cloud closer to the nucleus, also affecting the bond length (1.943 Å for C-OH bond and 1.400 Å for C-F bond) (see Supplementary Material B). Moreover, the F-C dipole is greater than the C-OH dipole. This depends both on the difference in electronegativity and the 3D geometry. The C-F bond is linear, while the C-OH bond shows a bent geometry. These differences lead to a marked change in ligand configuration comparing the 5-hydroxypentyl JWH-018 to AM-2201and could explain that the former act as partial agonist. All in all, the fluorine is both smaller and more polarized than the hydroxyl group. Addition of an alkene or a nitrile to the tail of the SC are other examples of modifications that retain both efficacy and potency and where the SC have been found to be involved in intoxications [40–42]. Both groups are planar, larger than fluorine but smaller in size and more electronegative than a hydroxyl group.

### Structure-activity comparison of hydroxyindole metabolites

The hydroxyindole metabolites of JWH-018 and AM-2201 varied in potency, efficacy and binding affinities. The overall hydrophobic interactions between the ligand and the hCB_1_ receptor were reduced for the inactive metabolites such as the 7-hydroxyindole metabolites. Both in silico and in vitro data indicate that monohydroxyindole metabolites originating from AM-2201 are more potent than those from JWH-018 (1.9–3.8 times), although they are less potent than their parent. This finding is in line with previous literature on the differences in potency of pentyl/5-fluoropentyl SC [43].

Our results show that all 4-hydroxy and 5-hydroxyindole metabolites retained more than 88.9% of the parent hCB_1_ efficacy. The 6-hydroxy and 7-hydroxyindole metabolites retained efficacy for AM-2201, but showed reduced efficacy for JWH-018. To the best of our knowledge, in vitro data on hCB_1_ receptor activity is available for only two hydroxyindole metabolites of JWH-018. Cannaert et al. reported that 6-hydroxyindole JWH-018 had significantly less *E*_max_ than its 5-hydroxy isomer [13]. The result of this study is consistent with the previous data.

Both 2-hydroxyindole metabolites were inactive in vitro and showed matching low predicted binding energies and *K*_i_, as well as fewer amino acid interactions in silico. The results of the SC metabolites tested in our study were consistent with the fact that all substructures of the molecule are involved in stabilizing the ligand in the hCB_1_ active site. Therefore, efficacy depended on the overall interactions within the hCB_1_ pocket rather than a single interaction. An example is seen when comparing the configurations of 7-hydroxyindole AM-2201 and 7-hydroxyindole JWH-018, where the 5-fluoro-pentyl tail stabilized the molecule in such a way that its terminal position after dynamics simulations was markedly different when compared to 7-hydroxyindole JWH-018 (Fig. 7).

### Implications, effects, and toxicity

To the best of our knowledge, this study is the first investigating potency of a large set of metabolites from a SC with the same in vitro hCB_1_ activity method and in combination with docking and molecular dynamics simulations. In total, 81% of the metabolites were found to activate the hCB_1_ receptor. The 4-hydroxypentyl, 5-hydroxypentyl and pentanoic acid metabolites are reported as major metabolites of JWH-018, AM-2201, THJ-018 and THJ-2201 [10, 44, 45]. In this study, the 4-hydroxypentyl metabolites were among the most potent metabolites, whilst acting as full agonists, whereas the 5-hydroxypentyl metabolites were less potent and were partial agonists. This finding suggests that metabolites contribute to the pharmacodynamic effects of SC and possibly prolonging their effects.

As previously mentioned, 5-hydroxypentyl JWH-018 is a metabolite whose structure can be formed from both JWH-018 and AM-2201, the latter undergoing defluorination during metabolism [45]. Similarly, 5-hydroxypentyl THJ-018 and the pentanoic acid metabolite of THJ-018 can be formed from both THJ-018 and THJ-2201 [44]. According to Wolfarth et al. 2015, there is a distinct difference in the metabolic patterns of SC with a pentyl tail versus a 5-fluoro pentyl tail, with the ratio of formed 4-hydroxypentyl/5-hydroxypentyl metabolites differing. During metabolism, SC with a pentyl tail seem to be predominantly hydroxylated at the fourth position over the fifth carbon of the pentyl tail [46]. Together with our results, this might indicate a possible prolonged effect of the pentyl tailed SC over the 5F-analogs. However, this needs to be confirmed by further studies as 5-hydroxypentyl metabolites are readily oxidized to the pentanoic acid via an aldehyde intermediate [47]. This metabolic route seems to be one of the major detoxification pathways to eliminate both pentyl and 5-flouro pentyl SC, as the pentanoic acids are inactive at the hCB_1_ receptor. To fully understand the pharmacological effect of the SC, other factors such as frequency of use, amount of dosage and clearance among other factors should also be considered.

In summary, we show that several prevalent phase I metabolites of JWH-018, AM-2201, THJ-018 and THJ-2201 activated the hCB_1_ receptor in vitro as agonists with efficacies and potencies comparable to the respective SC. Structure and activity relationship of positional isomers show that metabolic pathways resulting in 5-hydroxypentyl metabolites and pentanoic acid metabolites lead to a decrease in hCB_1_ activity, with the former acting as partial agonist and the latter being inactive. The efficacy data from in silico experiments correlated with the in vitro results demonstrating a linear trend between the binding affinity and efficacy of the compounds investigated. This correlation as well as the ability to explain the experimental data based on shifting binding poses validates the in silico model as a useful tool to model hCB1 binding to SC and their metabolites. Our data show that the efficacy and potency of the SC and their metabolites seem to be driven by a complex network of hydrophobic weak amino acid-ligand interactions. This study highlights that oxidation to 5-hydroxypentyl and the inactive pentanoic acid metabolites is likely an important mechanism for SC detoxification. In contrast, 4-hydroxypentyl metabolites retain both efficacy and potency and likely contribute to overall SC effects upon intake and possibly the duration of these cannabinergic effects. Additionally, the present study not only expound our understanding of SCRAs and their metabolites’ activity at the molecular level, but also presents a rapid and comprehensive model to enable clinical and forensic toxicologists, and public health advocates to respond timely to the constantly evolving and dynamic SCRA landscape, and NPS in general.

## Supplementary information

Supplementary Information

Supplementary material A

Supplementary material B

Supplementary figure1

Supplementary figure2

Supplementary figure3
