# Supplementary material for: Structure-activity relationship of prevalent synthetic cannabinoid metabolites on hCB1 in vitro and in silico dynamics
Source: Acta Pharmacol Sin. 2025 Nov 3;47(3):776–89. doi: 10.1038/s41401-025-01678-5 (PMC12932713; doi:10.1038/s41401-025-01678-5)
Supplement: Supplementary file 2 — Supplementary material A [file 41401_2025_1678_MOESM2_ESM.pdf]

Supplementary material A

In vitro structure relationship activity and molecular dynamic simulation studies of JWH-018, AM-2201, THJ-018, THJ-2201 and their metabolites on the hCB_1 receptor

Anna Åstrand[a], Emiliano Laudadio[b], Prince S. Gameli[c], Laura Martin[d], Jeremy Carlier[c], Francesco P. Busardo[c], Johan Dahlén[e], Xiongyu Wu[e], Peter Konradsson[e], Svante Vikingsson[a,f,g], Robert Kronstrand[a,f] and Henrik Green[a,f]

[a] Division of Drug Research, Department of Medical and Health Sciences, Faculty of Medicine and Health Sciences, Linköping University, SE 581 85 Linköping, Sweden

[b] Department of Science and Engineering of Matter, Environment and Urban Planning, Polytechnic University of Marche, Ancona, Italy

[c] Department of Biomedical Sciences and Public Health, Polytechnic University of Marche, Ancona, Italy

[d] Institute of Chemistry and Bioanalytics, School of Life Sciences, University of Applied Sciences and Arts Northwestern Switzerland, CH 4132 Muttenz, Switzerland

[e] Department of Physics, Chemistry and Biology, Linköping University, Linköping, Sweden

[f] Department of Forensic Genetics and Forensic Toxicology, National Board of Forensic Medicine, SE 587 58 Linköping, Sweden

[g] Center for Forensic Science Advancement and Application, RTI International, 3040 East Cornwallis Rd., Research Triangle Park, NC, 27709, USA

Corresponding author:

Anna Åstrand

Division of Clinical Chemistry and Pharmacology

Department of Biomedical and Clinical Sciences

Linköping University

SE-581 83 Linköping

Sweden

Mail: anna.astrand@liu.se

Background Information of Synthetic Cannabinoid Metabolite SCM-052

Linköping University
INSTITUTE OF TECHNOLOGY

Contents

Substance Information 3
Synthetic Scheme 4
NMR Spectra 5
General Information 5
^{1}H-NMR Spectrum 5
^{13}C-NMR Spectrum 7
Liquid Chromatography 8
General Information 8
UV-Vis Chromatogram 8

Substance Information

Chemical Name:	*(1-(5-fluoro-4-hydroxypentyl)-1H-indazol-3-yl)(naphthalen-1-yl)methanone*
Chemical Formula:	*$C_{23}H_{21}FN_2O_2$*
Serial Number:	*SCM-052*
Molecular Structure:	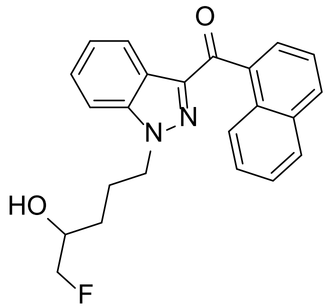
Molecular Weight:	*376.43 g/mol*
Rf-value:	*0.40 (1:1 EtOAc/n-Hep)*
Date of Completion:	*2016-02-05*
Amount:	*5.0 mg*
Purity:	*>98%*

Synthetic Scheme

Not applicable

NMR Spectra

General Information

^{1}H, ^{19}F and ^{13}C spectra were recorded on a Varian Mercury 300 MHz instrument at 25^{0}C in $CDCl_3$.

^{1}H-NMR Spectrum

Solvent: $CDCl_3$

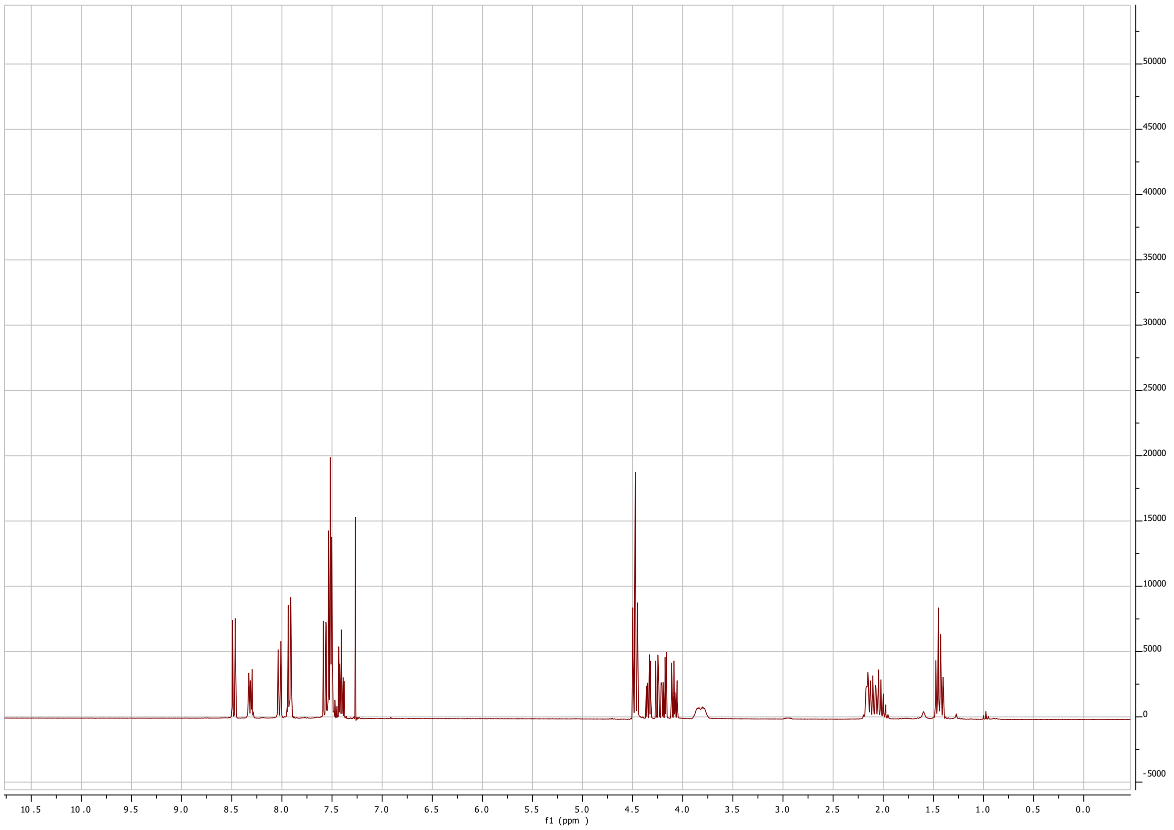

^{19}F NMR ($CDCl_3$)

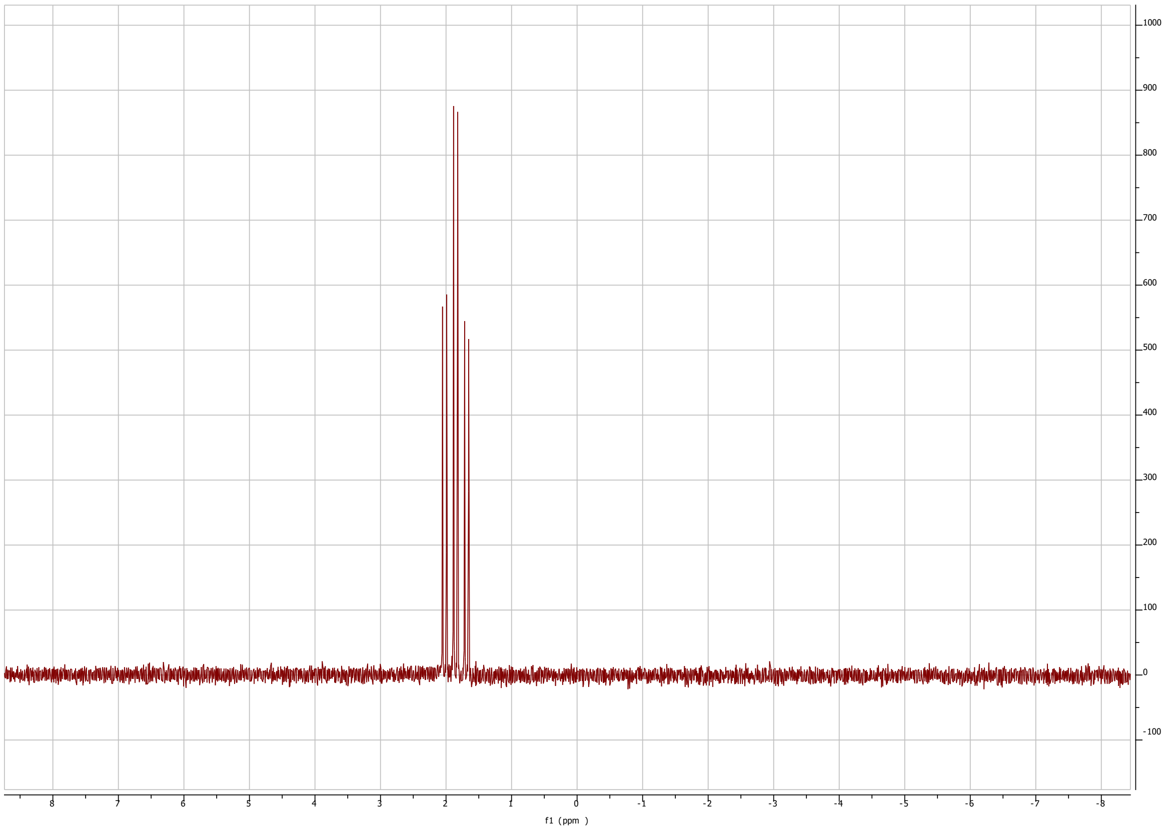

^{13}C-NMR Spectrum

Solvent: $CDCl_3$

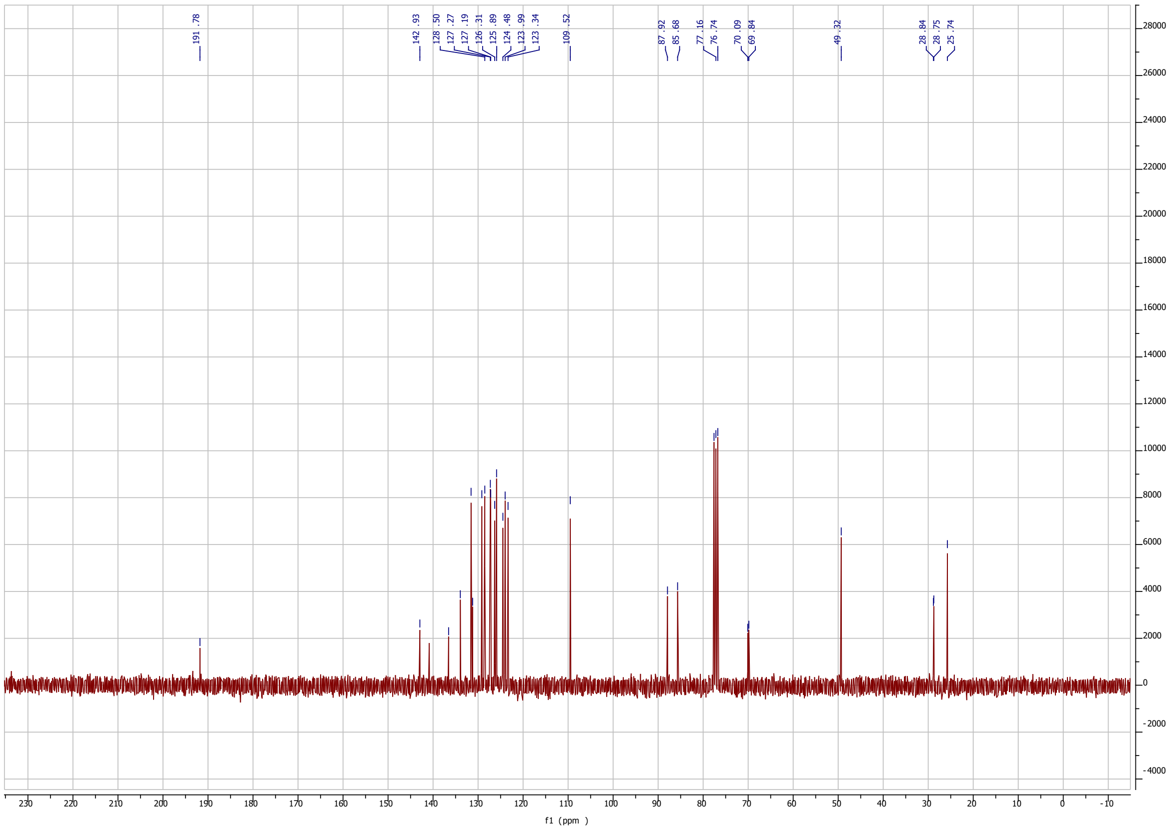

Liquid Chromatography

General Information

Column: Xbridge C18, 2.5 uM, 4.6 x 50 mm.

Mobile phase system: 25:75 to 90:0 / B:A (B: 90:10 / acetonitril:water, 10 mM NH_4OAc; A: 5:95 / acetonitrile:water, 10 mM NH_4OAc

Mobile phase program: Time1 = 5 min, Time2 = 2 min, Flow = 2 ml/min,
λ = 215 nm

UV-Vis Chromatogram

Solvent: Methanol

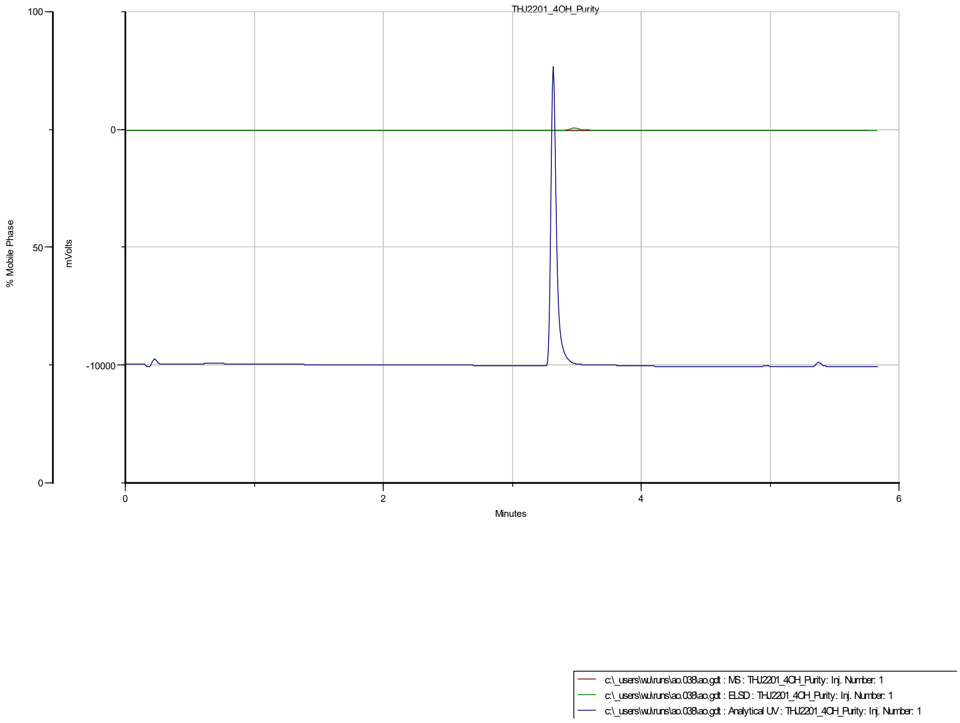

Purity:

Retention time (min)	Peak area	Percentage (%)
**3.32**	66866184	98.6
**5.38**	930846	1.4

Background Information of Synthetic Cannabinoid Metabolite SCM-060

Linköping University
INSTITUTE OF TECHNOLOGY

Contents

Substance Information 3
Synthetic Scheme 4
NMR Spectra 5
General Information 5
^{1}H-NMR Spectrum 5
^{19}F NMR ($CDCl_3$) 6
^{13}C-NMR Spectrum 7
Liquid Chromatography 8
General Information 8
UV-Vis Chromatogram 8
Mass spectrum 9

Substance Information

Chemical Name:	*(1-(5-fluoro-3-hydroxypentyl)-1H-indazol-3-yl)(naphthalen-1-yl)methanone*
General Name:	*3-OH-THJ2201*
Chemical Formula:	$C_{23}H_{22}FN_2O_2$
Serial Number:	*SCM-060*
Molecular Structure:	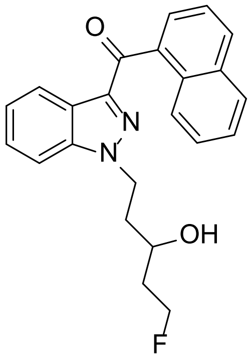
Molecular Weight:	*376.42 g/mol*
Rf-value:	-
Date of Completion:	*2017-06-16*
Amount:	*450 uL (Concentration: 0.3 mg in 500 uL MeOH)*
Purity:	-

Synthetic Scheme

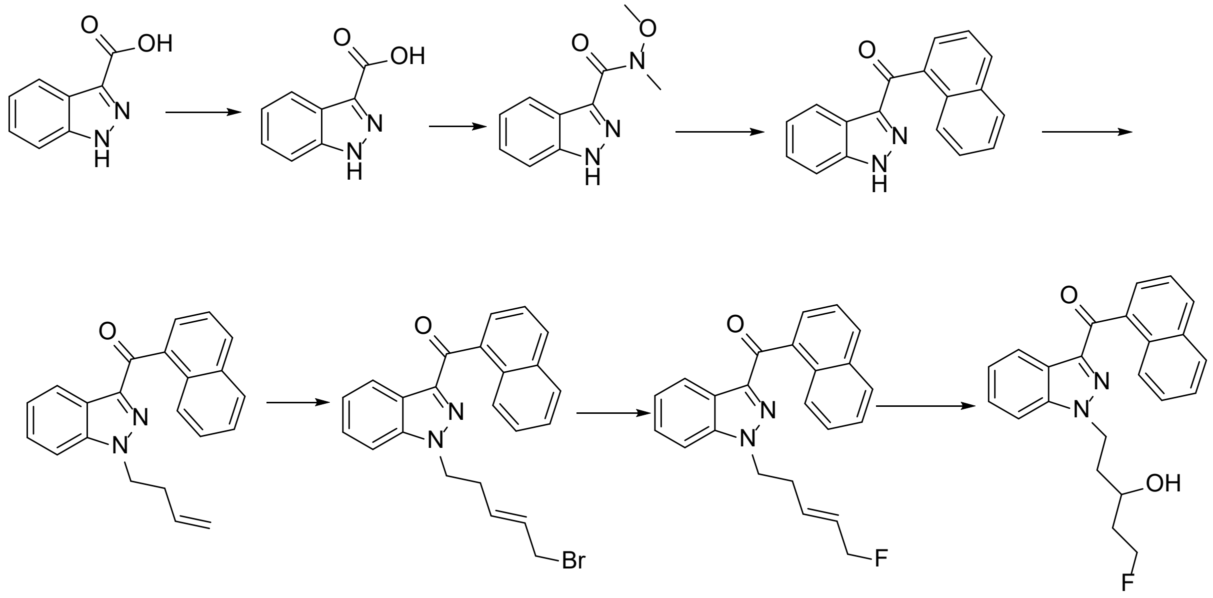

NMR Spectra

General Information

^{1}H, ^{19}F and ^{13}C spectra were recorded on a Varian Mercury 300 MHz instrument at 25^{0}C in $CDCl_3$.

^{1}H-NMR Spectrum

Solvent: $CDCl_3$

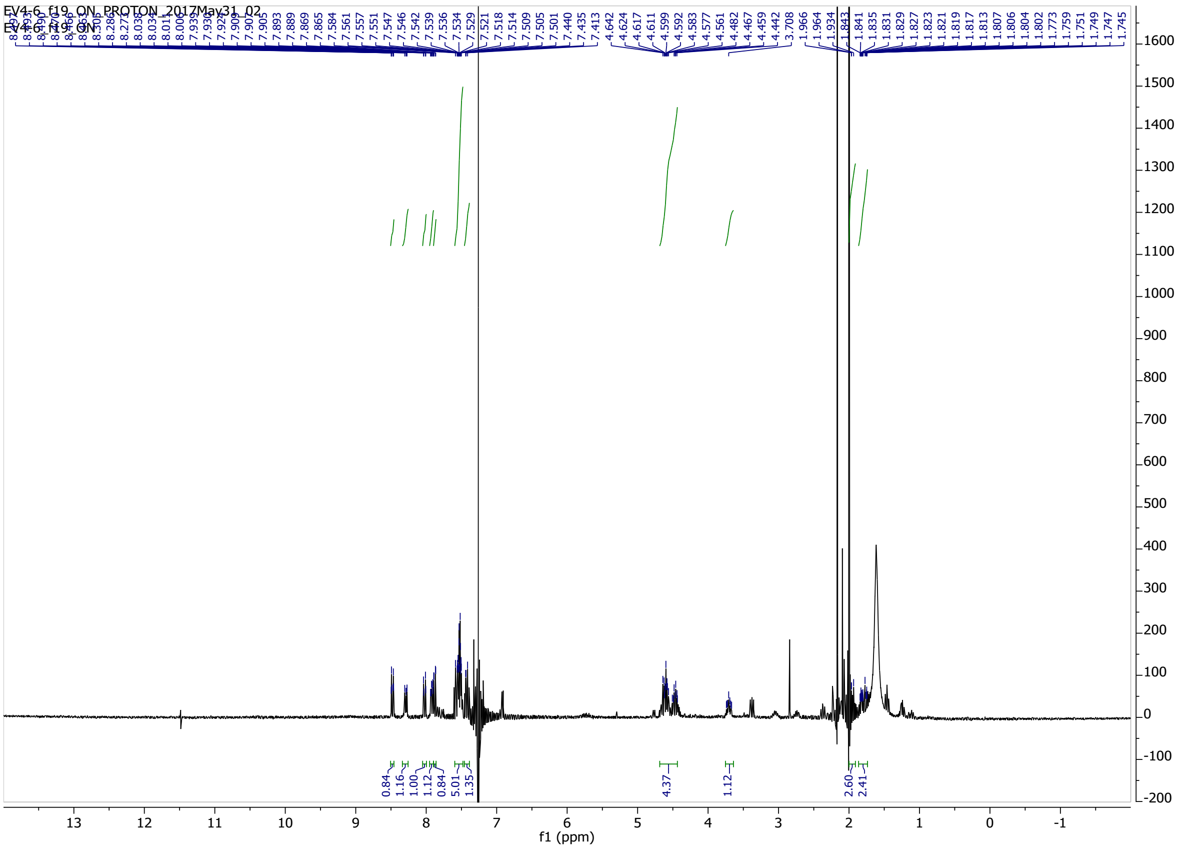

^{19}F NMR ($CDCl_3$)

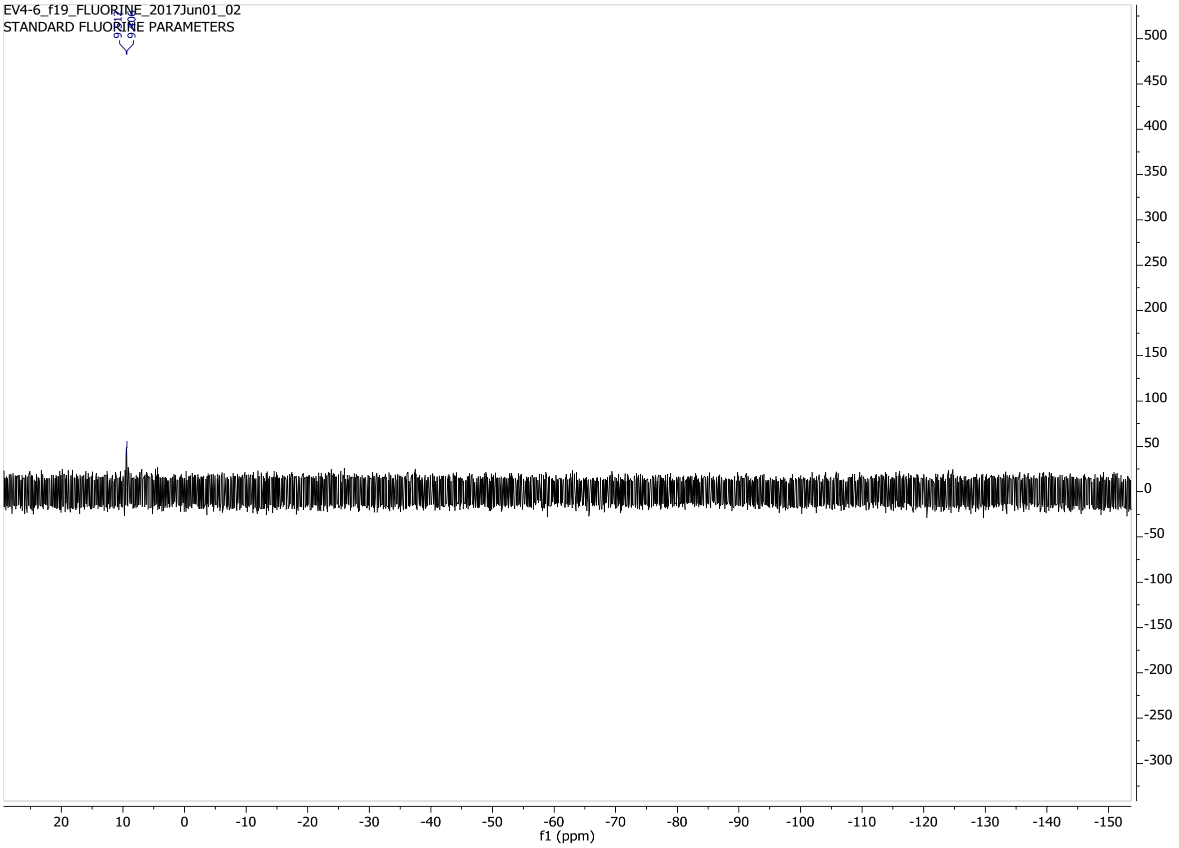

^{13}C-NMR Spectrum

Solvent: $CDCl_3$

Not available

Liquid Chromatography

General Information

Column: Xbridge C18, 2.5 uM, 4.6 x 50 mm.

Mobile phase system: 20:80 to 100:0 / B:A (B: 90:10 / acetonitril:water, 10 mM NH_4OAc; A: 5:95 / acetonitrile:water, 10 mM NH_4OAc

Mobile phase program: Gradient Time = 5 min, Hold Time = 1 min, Flow = 1.5 ml/min,

UV-Vis Chromatogram

Solvent: Acetonitrile

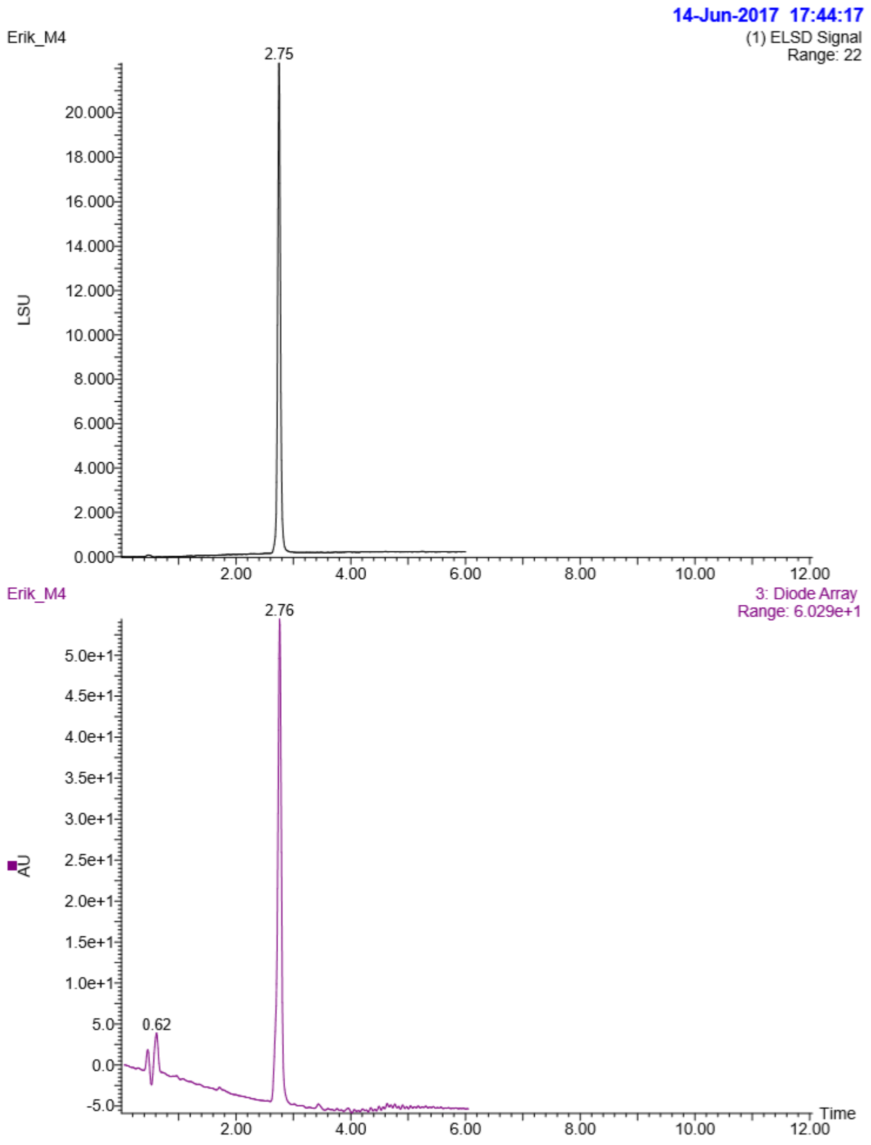

Mass spectrum (ESI$^+$)

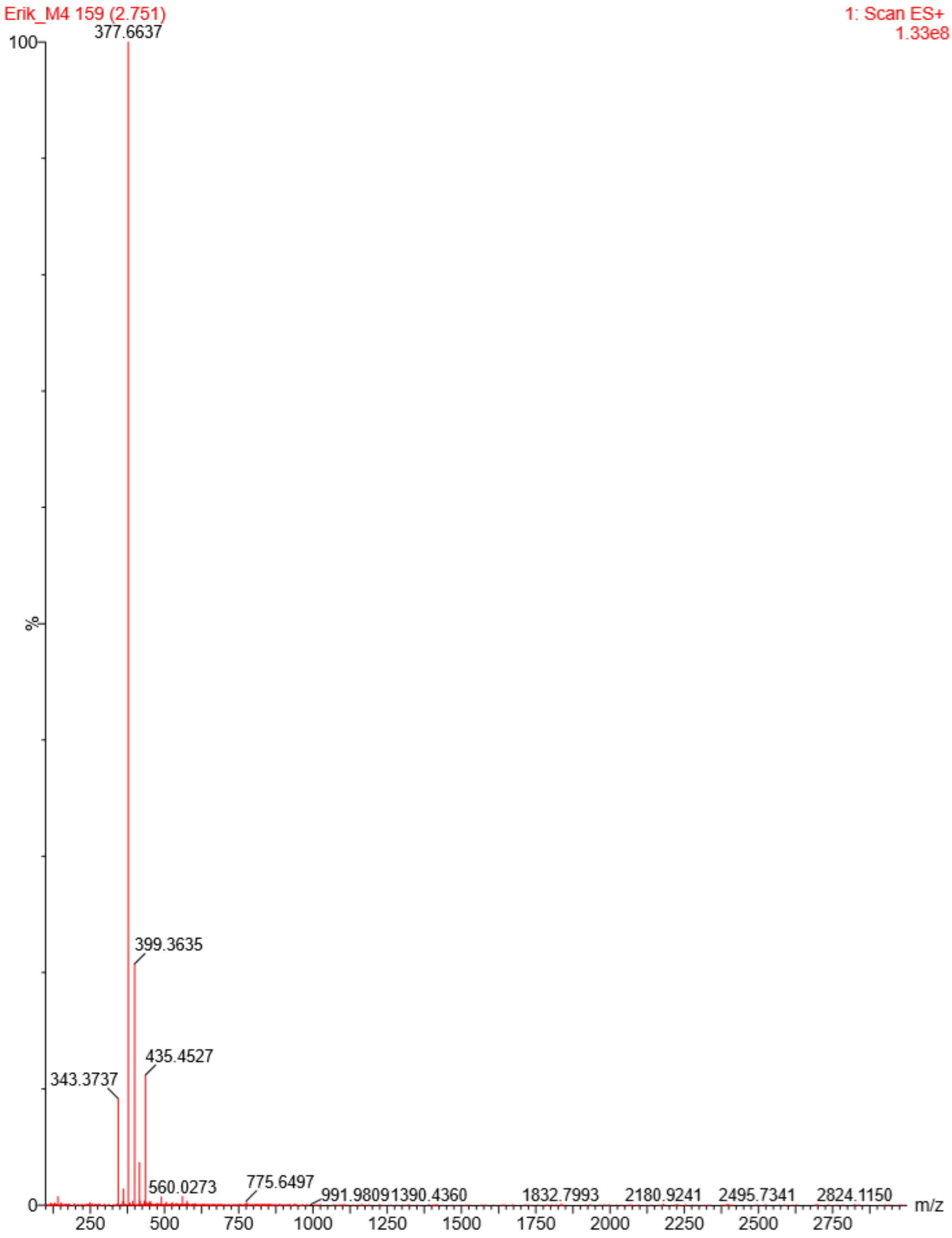

Background Information of Synthetic Cannabinoid Metabolite SCM-059

Linköping University
INSTITUTE OF TECHNOLOGY

Contents

Substance Information 3
Synthetic Scheme 4
NMR Spectra 5
General Information 5
^{1}H-NMR Spectrum 5
^{19}F NMR ($CDCl_3$) 6
^{13}C-NMR Spectrum 7
Liquid Chromatography 8
General Information 8
UV-Vis Chromatogram 8
Mass spectrum 9

Substance Information

Chemical Name:	*(1-(5-fluoro-2-hydroxypentyl)-1H-indazol-3-yl)(naphthalen-1-yl)methanone*
General Name:	*2-OH-THJ2201*
Chemical Formula:	$C_{23}H_{22}FN_2O_2$
Serial Number:	*SCM-059*
Molecular Structure:	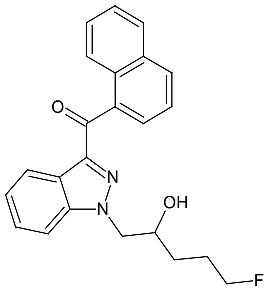
Molecular Weight:	*376.42 g/mol*
Rf-value:	-
Date of Completion:	*2017-02-16*
Amount:	*3.3 mg*
Purity:	-

Synthetic Scheme

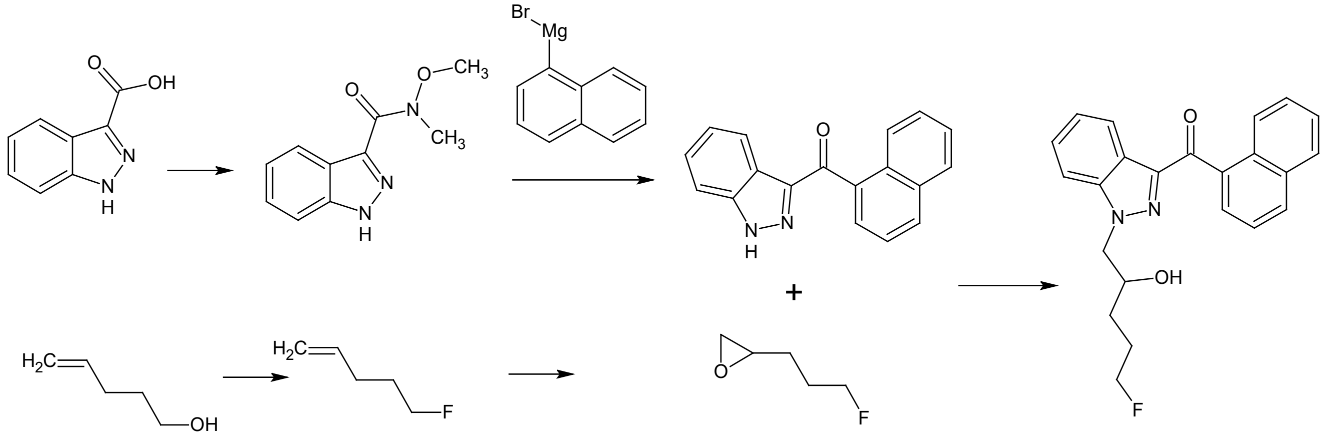

NMR Spectra

General Information

^{1}H, ^{19}F and ^{13}C spectra were recorded on a Varian Mercury 300 MHz instrument at 25^{0}C in $CDCl_3$.

^{1}H-NMR Spectrum

Solvent: $CDCl_3$

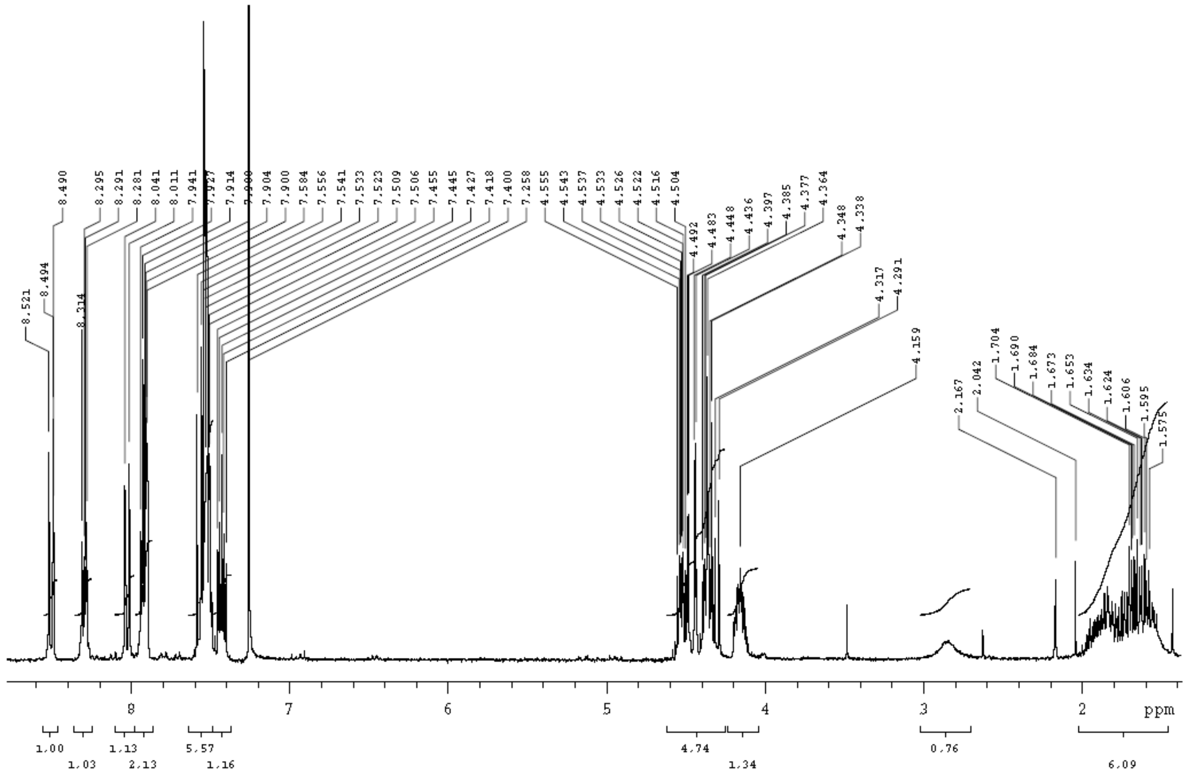

^{19}F NMR ($CDCl_3$)

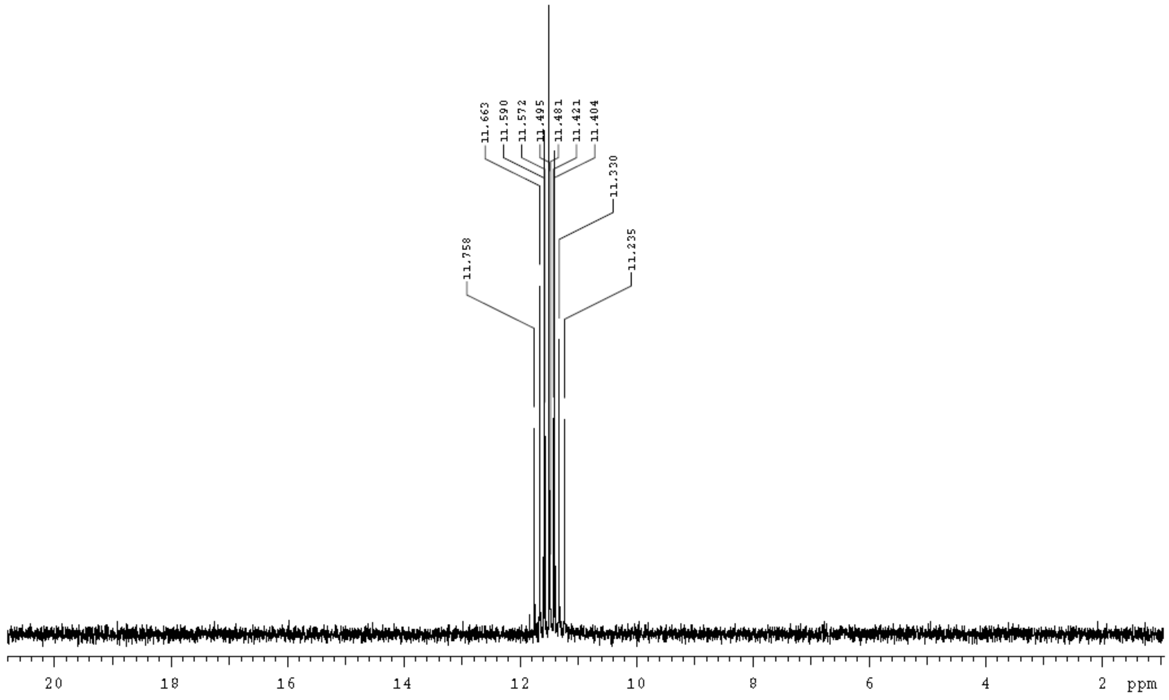

^{13}C-NMR Spectrum

Solvent: $CDCl_3$

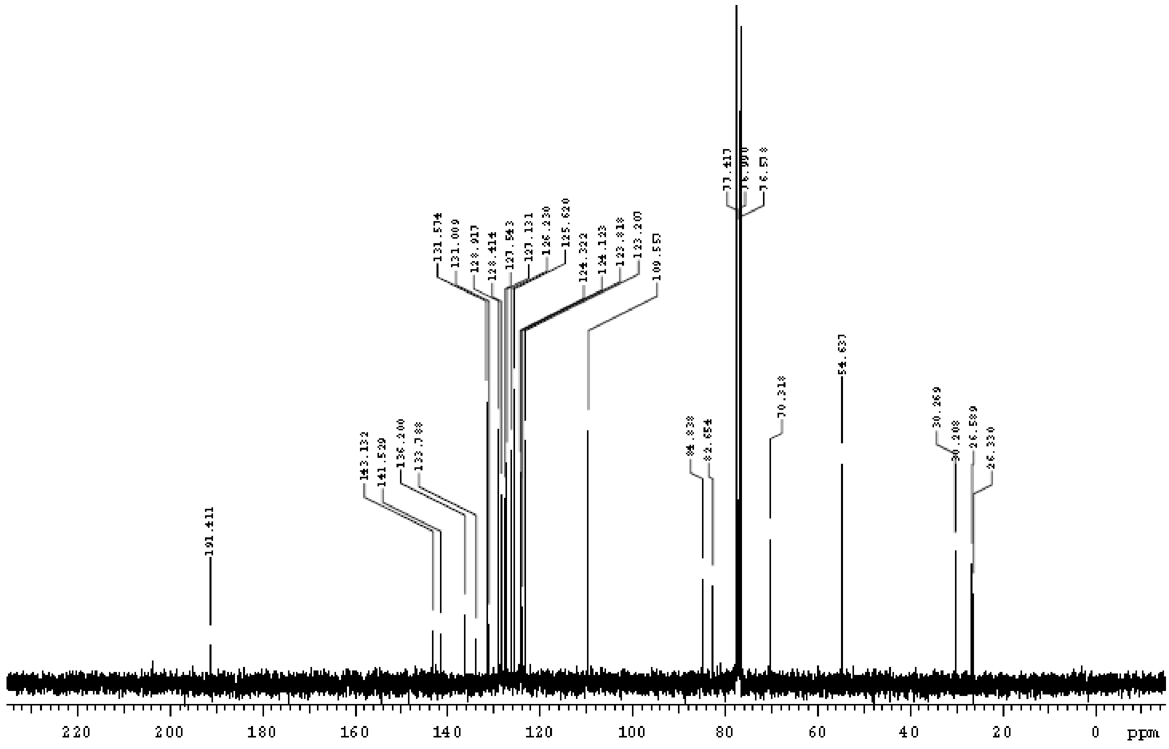

Liquid Chromatography

General Information

Column: Xbridge C18, 2.5 uM, 4.6 x 50 mm.

Mobile phase system: 20:80 to 100:0 / B:A (B: 90:10 / acetonitril:water, 10 mM NH_4OAc; A: 5:95 / acetonitrile:water, 10 mM NH_4OAc

Mobile phase program: Gradient Time = 5 min, Hold Time = 1 min, Flow = 1.5 ml/min,

UV-Vis Chromatogram

Solvent: Acetonitrile

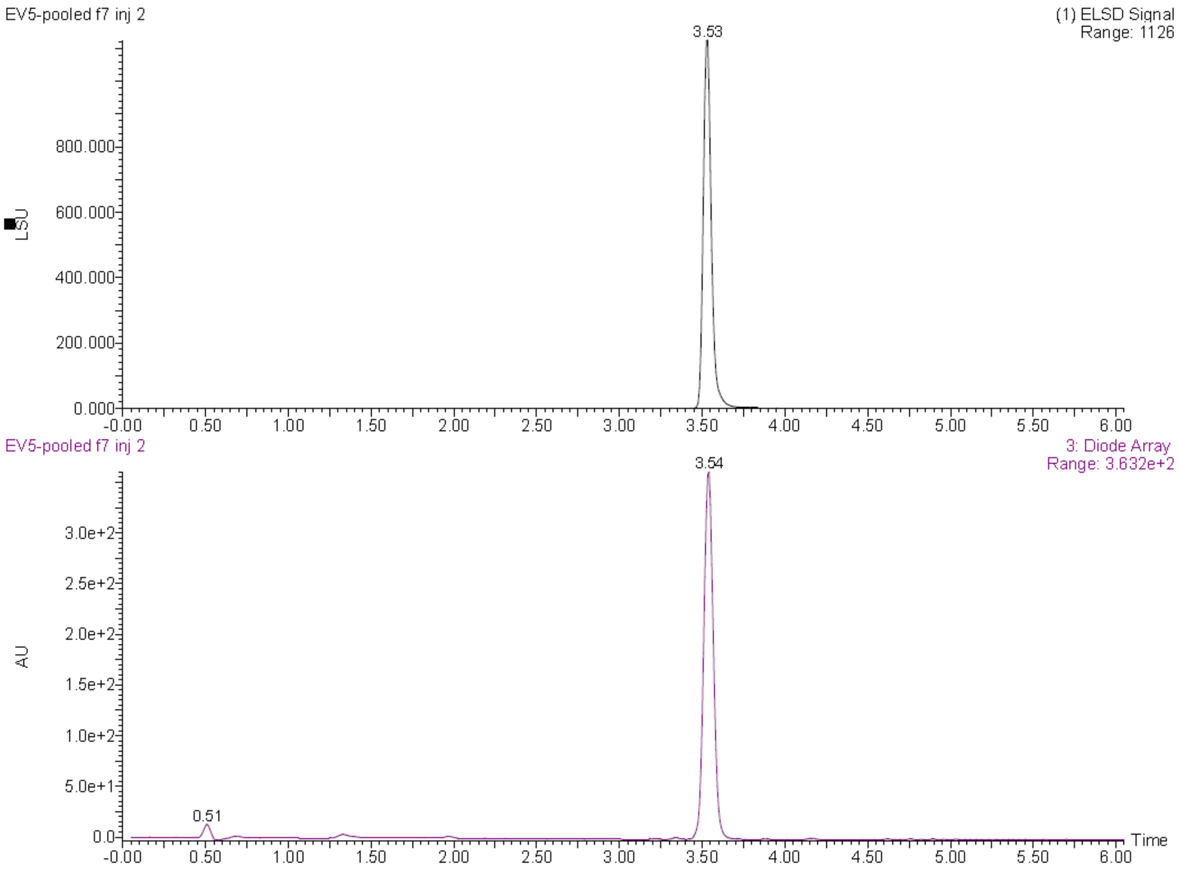

Mass spectrum (ESI$^+$)

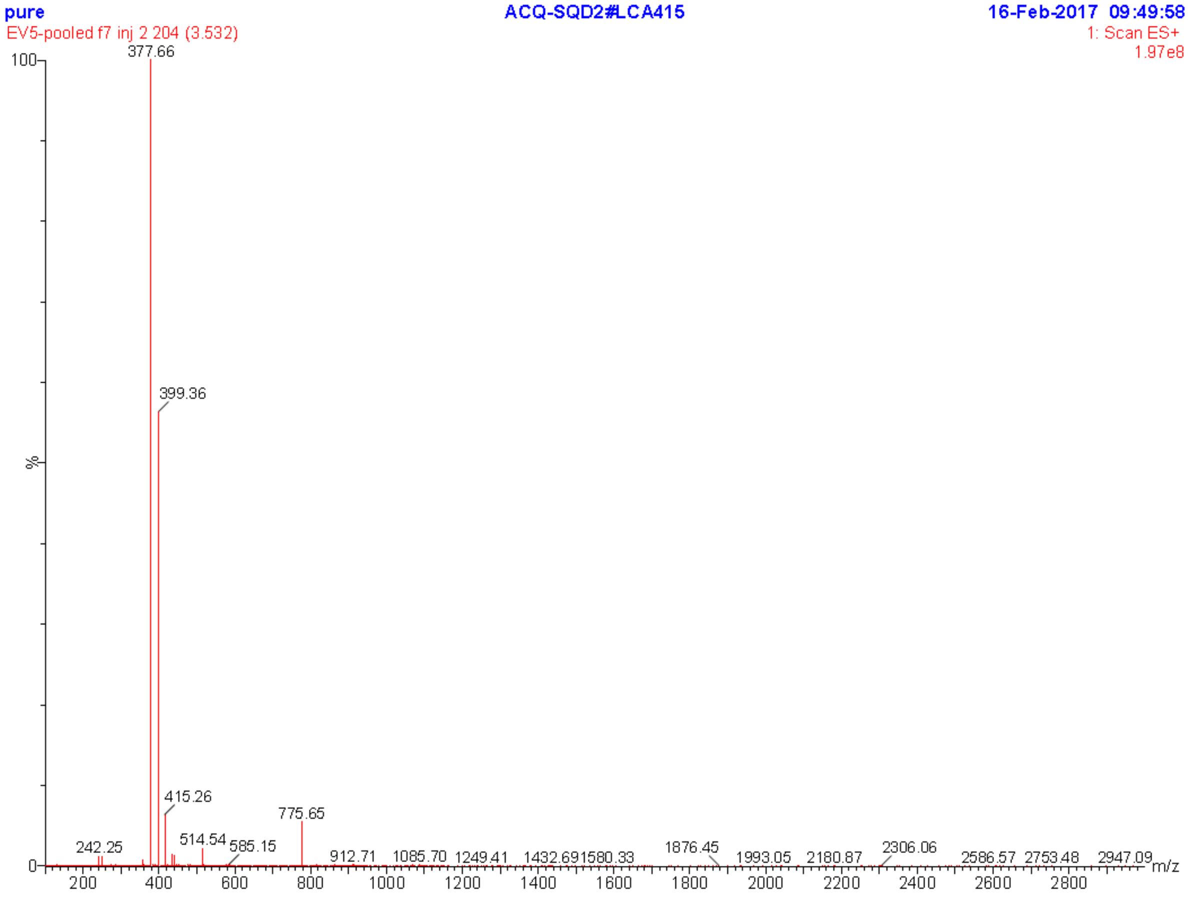

Background Information of Synthetic Cannabinoid Metabolite SCM-049

Linköping University

INSTITUTE OF TECHNOLOGY

Contents

Substance Information 3
Synthetic Scheme 4
NMR Spectra 5
General Information 5
^{1}H-NMR Spectrum 5
^{13}C-NMR Spectrum 6
Liquid Chromatography 7
General Information 7
UV-Vis Chromatogram 7
Mass Spectrum 8

Substance Information

Chemical Name:	*(1-(4-hydroxypentyl)-1H-indazol-3-yl)(naphthalen-1-yl)methanone*
Chemical Formula:	$C_{23}H_{22}N_2O_2$
Serial Number:	*SCM-049*
Molecular Structure:	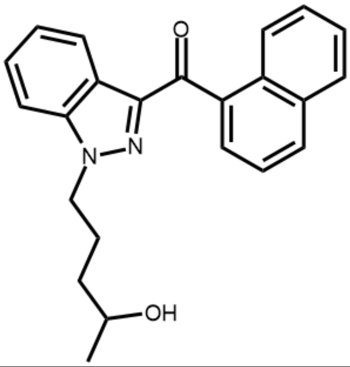
Molecular Weight:	*358.44 g/mol*
Rf-value:	-
Date of Completion:	*2016-02-05*
Amount:	*0.8 mg*
Comments:	-

Synthetic Scheme

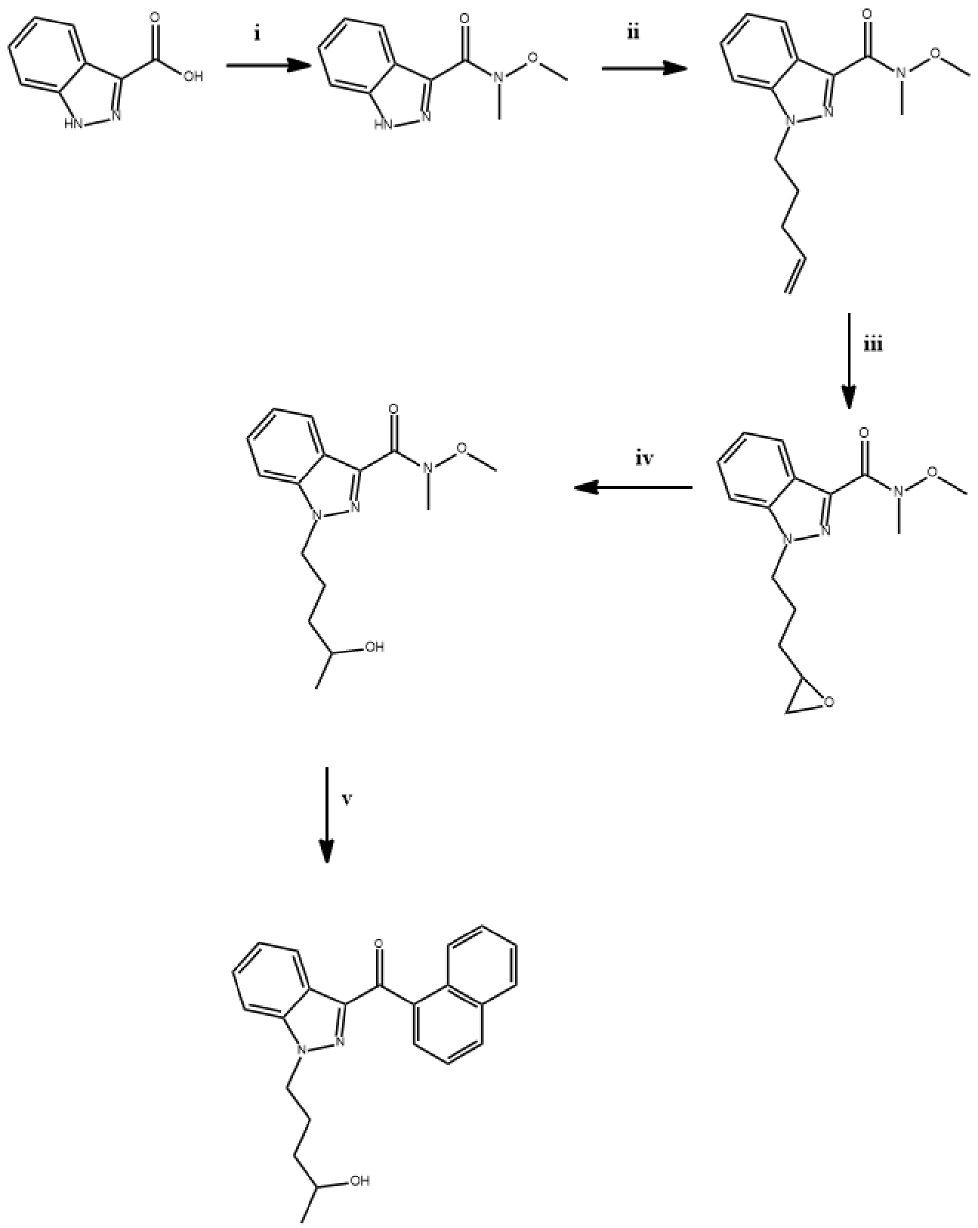

i) N,O-dimethylhydroxylamine hydrochloride, Pyridine, EDC, THF; ii) NaH, DMF, 5-bromo-1-pentene; iii) mCPBA, DCM; iv) $NaBH_4$, MeOH; V) #1. $Mg_{(s)}$, 1-Bromonaphthalene, I_2, dry THF, MW: 40 min. 100 °C. #2.MW: 10 min. 80 °C + 15h, rt, N_2. #3. NH_4Cl;

NMR Spectra

General Information

^{1}H, ^{13}C and ^{19}F-NMR spectra were recorded on Varian Mercury 300 MHz instrument at 25^{0}C in $CDCl_3$, MeOH-d_4 or acetone-d_6.

^{1}H-NMR Spectrum

Solvent: $CDCl_3$

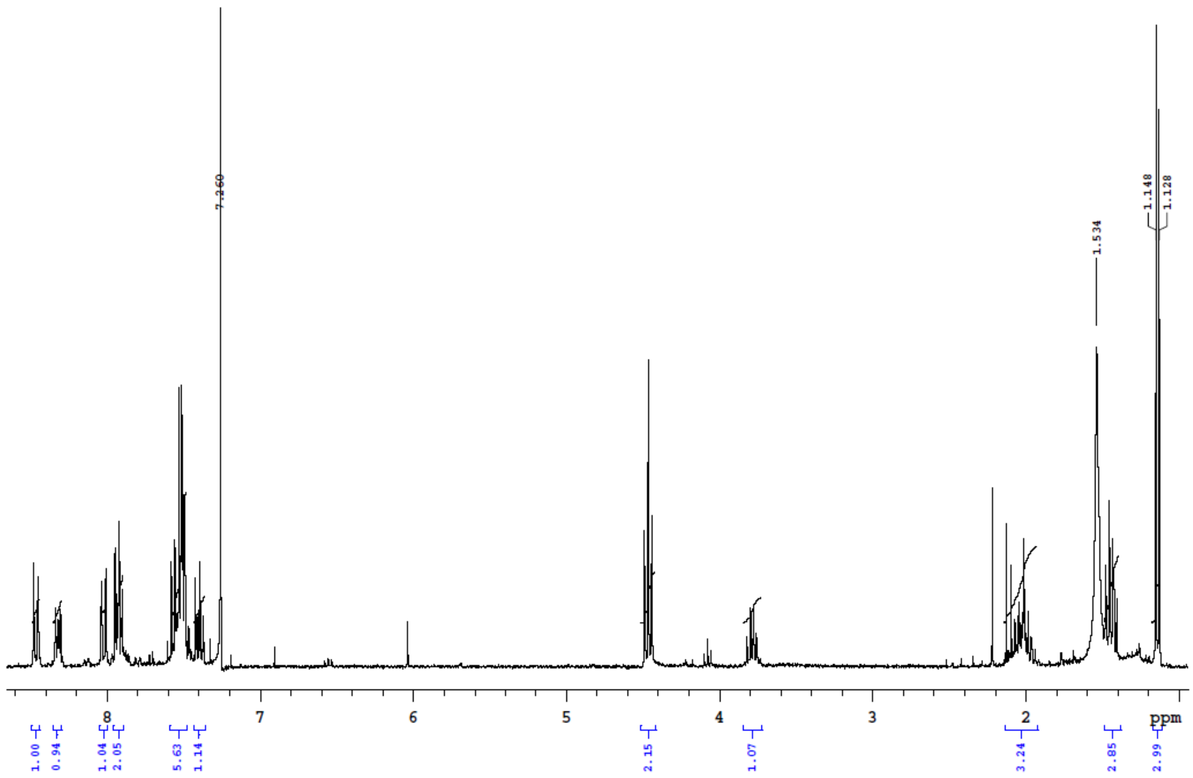

^{13}C-NMR Spectrum

Solvent: $CDCl_3$

Not available

Liquid Chromatography

General Information

Column: C8, 2.5 µm, 4.6 x 50 mm.

Mobile phase system: ACN/H_2O

UV-Vis Chromatogram

Solvent: ACN

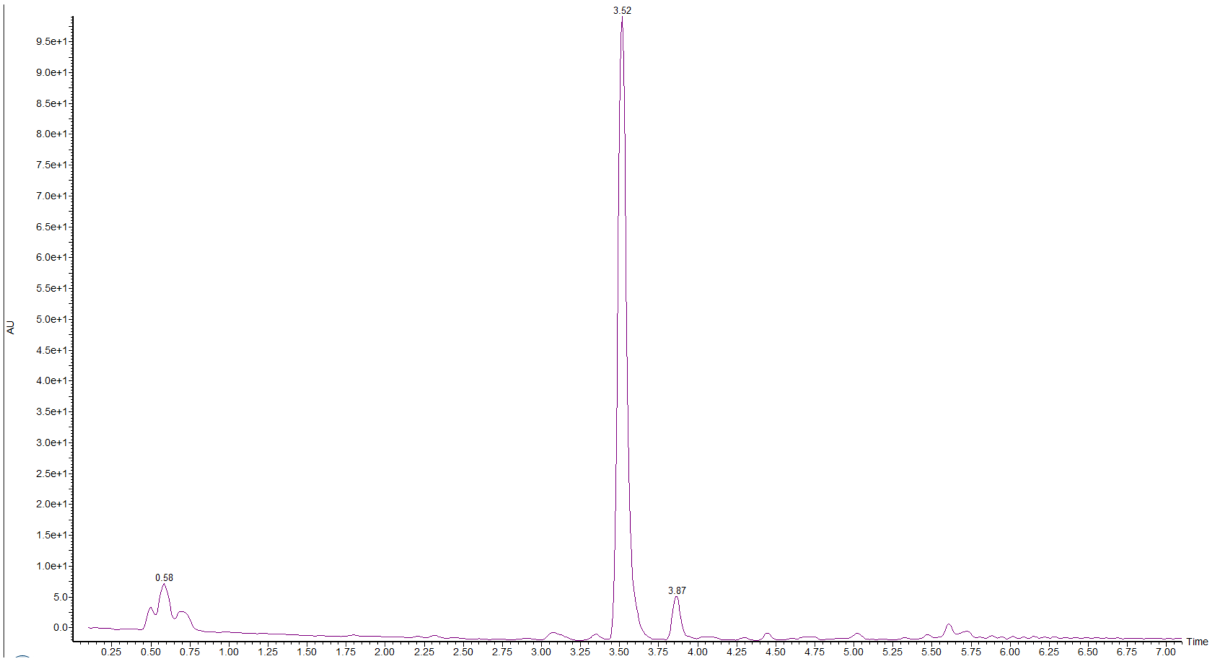

Mass Spectrum

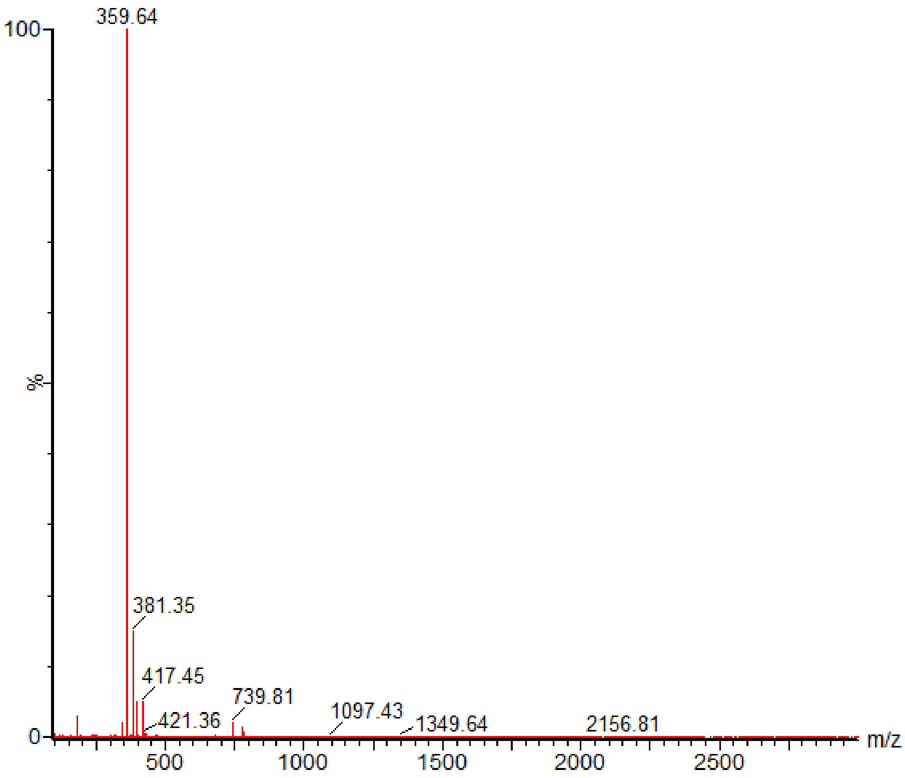

Background Information of Synthetic Cannabinoid Metabolite SCM-054

LINKÖPINGS UNIVERSITET

Linköping University

INSTITUTE OF TECHNOLOGY

Contents

Substance Information 3
Synthetic Scheme 4
NMR Spectra 5
General Information 5
^{1}H-NMR Spectrum 5
^{19}F NMR ($CDCl_3$) 6
^{13}C-NMR Spectrum 7
Liquid Chromatography 8
General Information 8
UV-Vis Chromatogram 8
Mass spectrum 9

Substance Information

Chemical Name:	*(1-(5-fluoro-2-hydroxypentyl)-1H-indol-3-yl)(naphthalen-1-yl)methanone*
Chemical Formula:	$C_{24}H_{22}FNO_2$
Serial Number:	*SCM-054*
Molecular Structure:	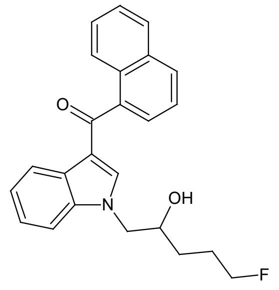
Molecular Weight:	*375.43 g/mol*
Rf-value:	-
Date of Completion:	*2017-01-31*
Amount:	*5 mg*
Purity:	-

Synthetic Scheme

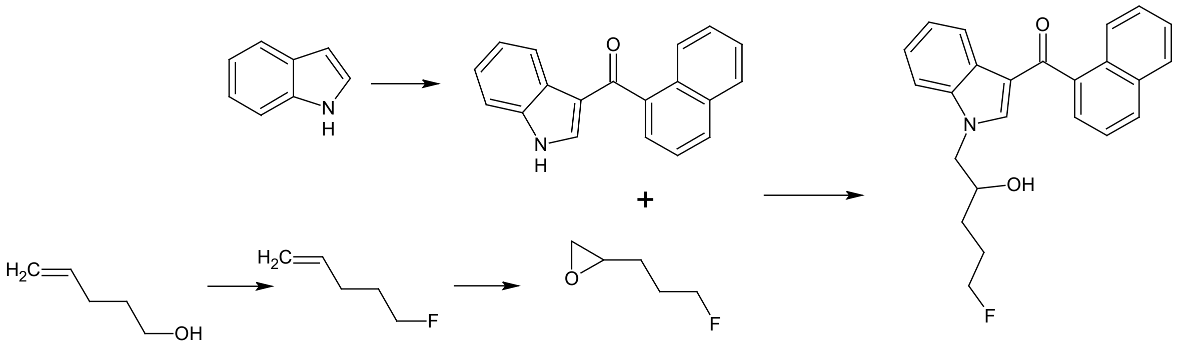

NMR Spectra

General Information

^{1}H, ^{19}F and ^{13}C spectra were recorded on a Varian Mercury 300 MHz instrument at 25^{0}C in $CDCl_3$.

^{1}H-NMR Spectrum

Solvent: $CDCl_3$

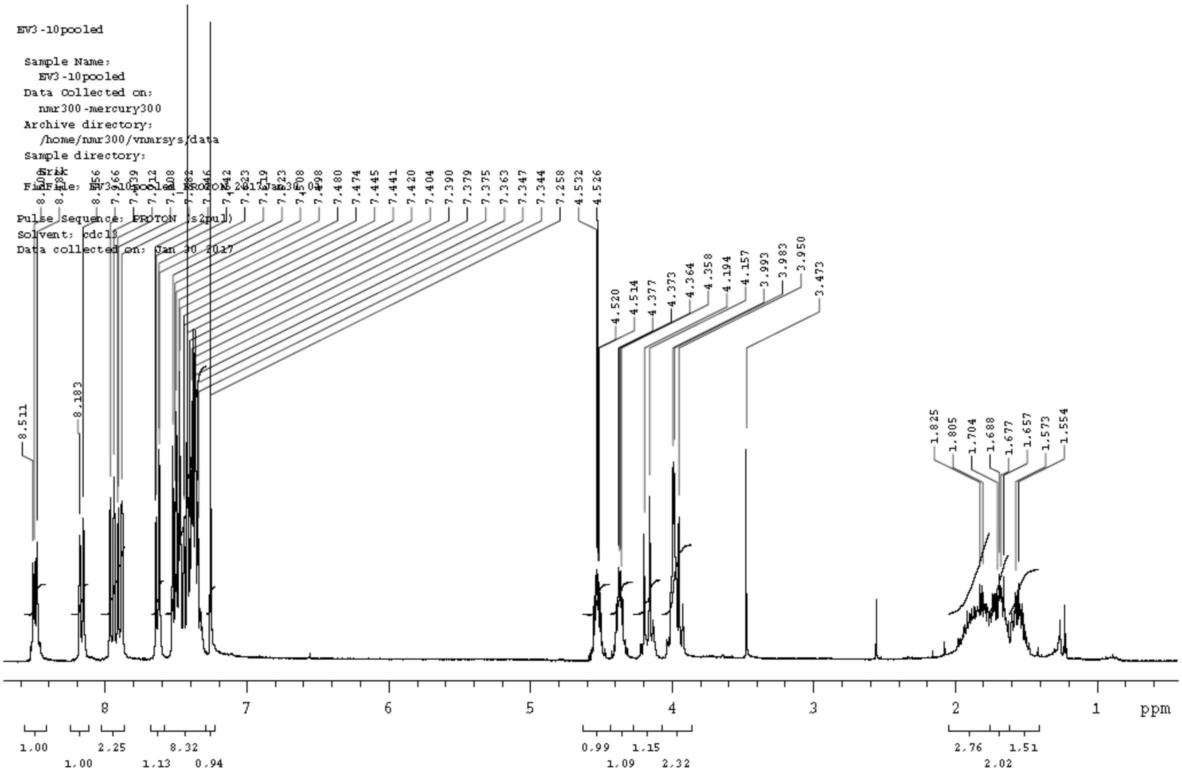

^{19}F NMR ($CDCl_3$)

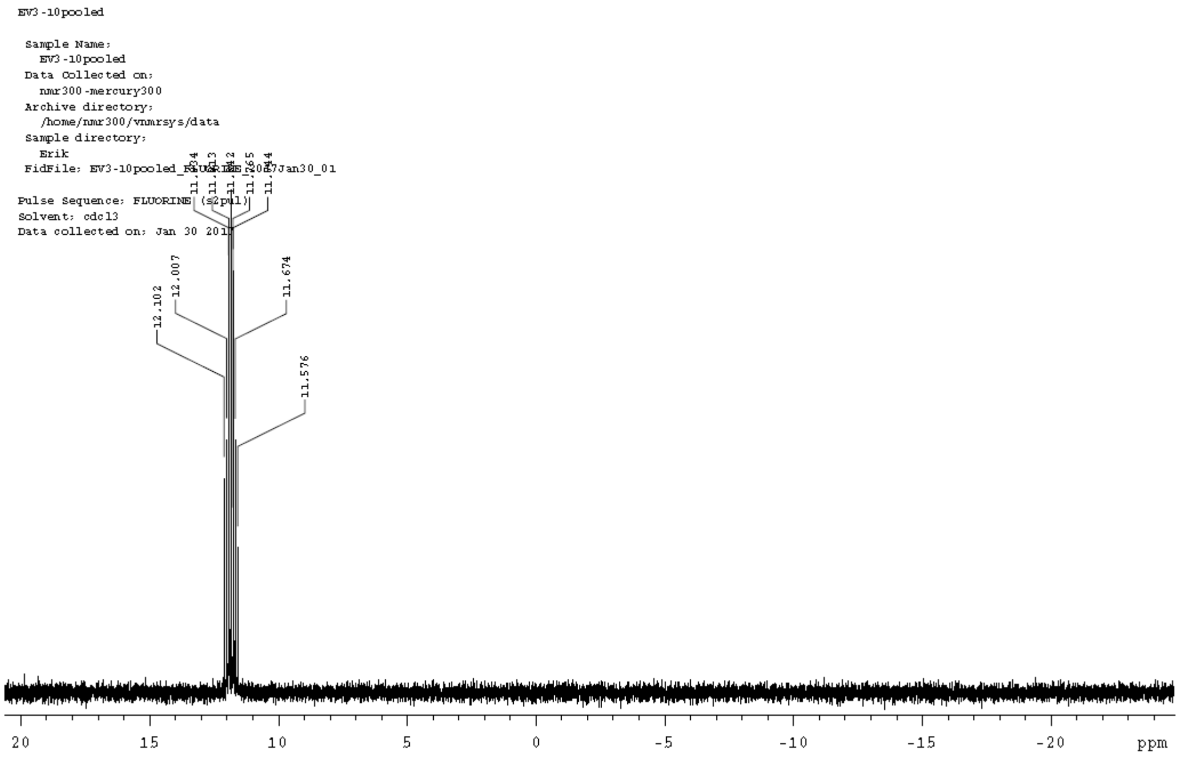

^{13}C-NMR Spectrum

Solvent: $CDCl_3$

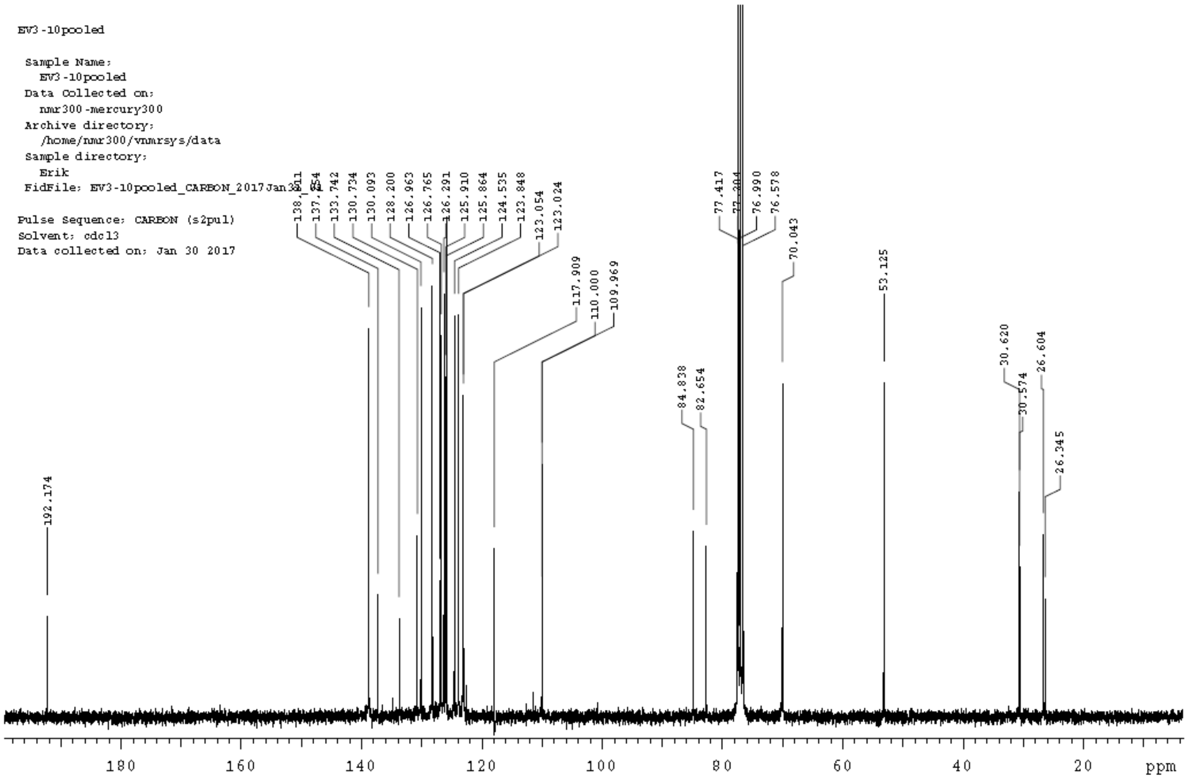

Liquid Chromatography

General Information

Column: Xbridge C18, 2.5 uM, 4.6 x 50 mm.

Mobile phase system: 20:80 to 100:0 / B:A (B: 90:10 / acetonitril:water, 10 mM NH_4OAc; A: 5:95 / acetonitrile:water, 10 mM NH_4OAc

Mobile phase program: Time1 = 5 min, Time2 = 1 min, Flow = 1.5 ml/min,

UV-Vis Chromatogram

Solvent: Acetonitrile

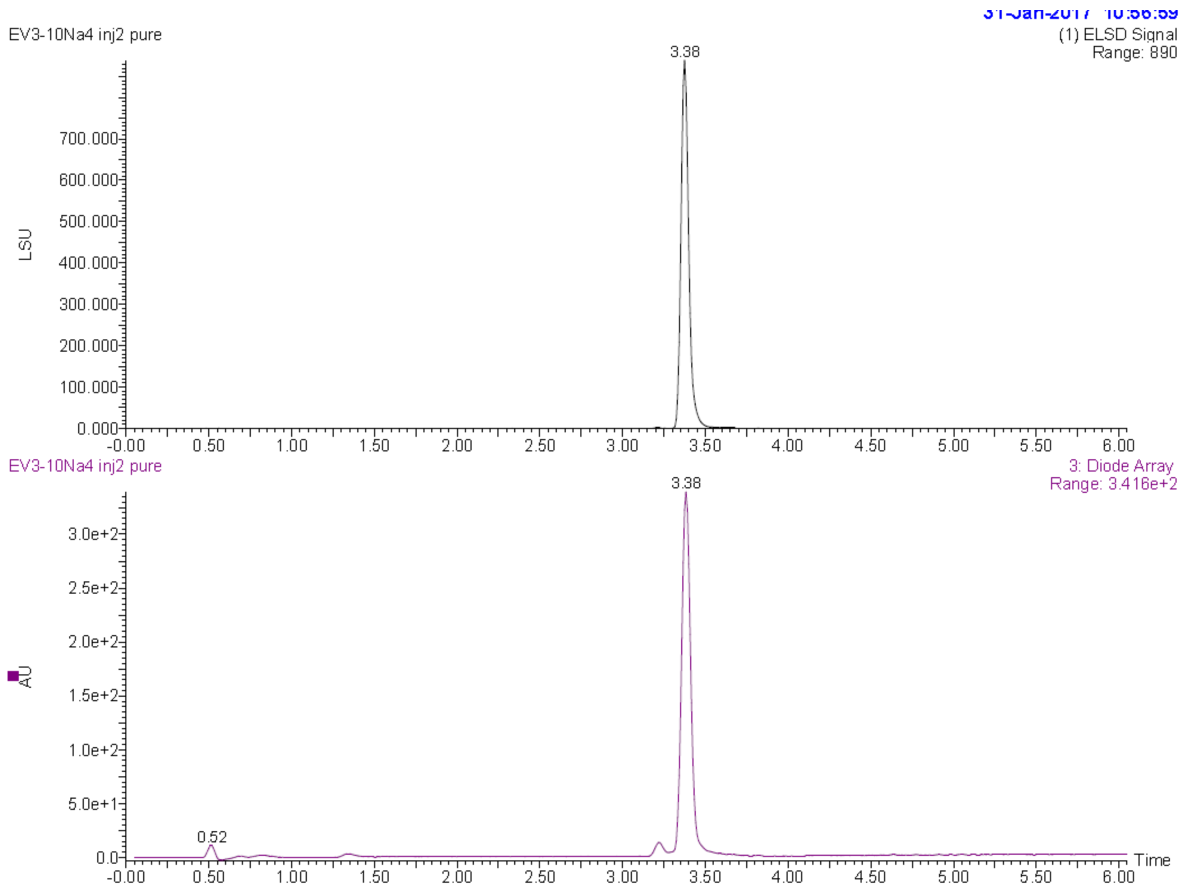

Mass spectrum

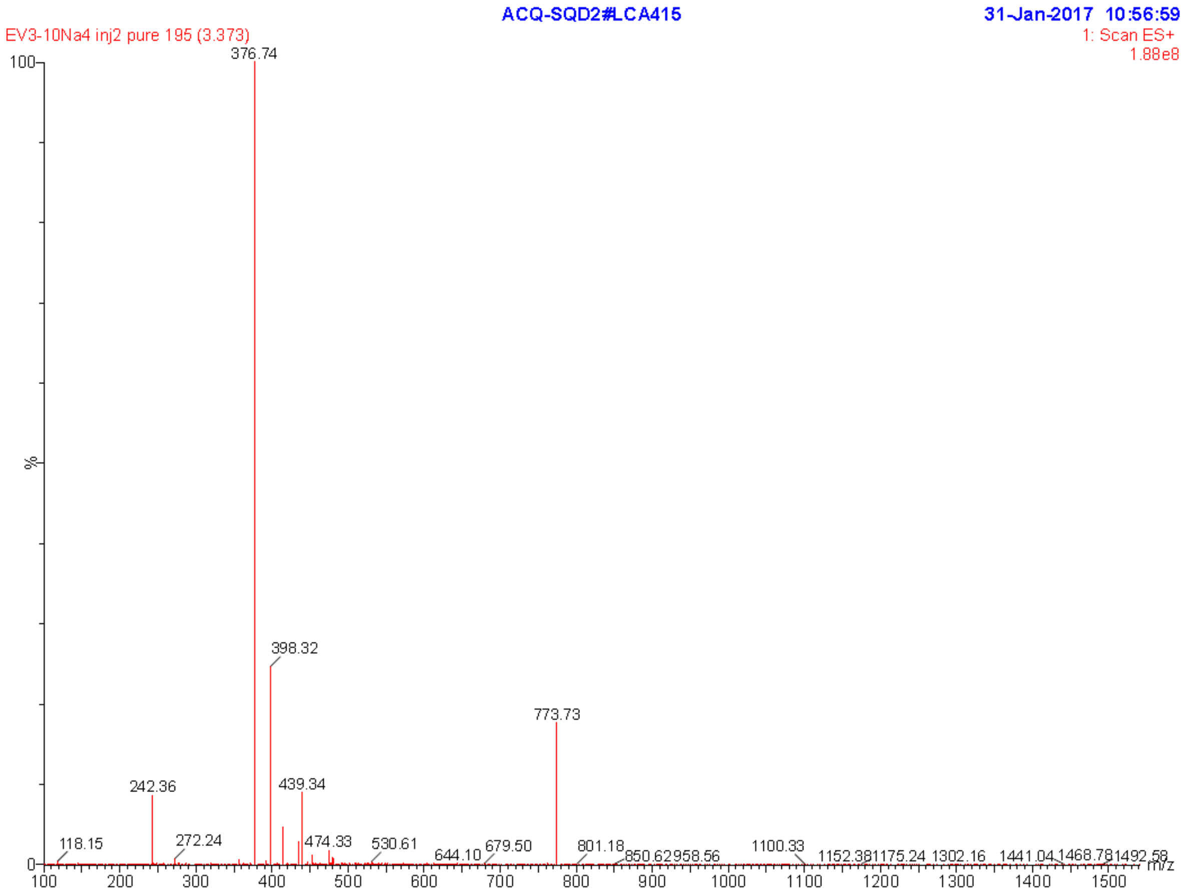

(1-(2-hydroxypentyl)-1H-indol-3-yl)(naphthalen-1-yl)methanone, JWH-018 2-hydroxypentyl (SCM-002) and (1-(3-hydroxypentyl)-1H-indol-3-yl)(naphthalen-1-yl)methanone JWH-018 3-hydroxypentyl (SCM-003)

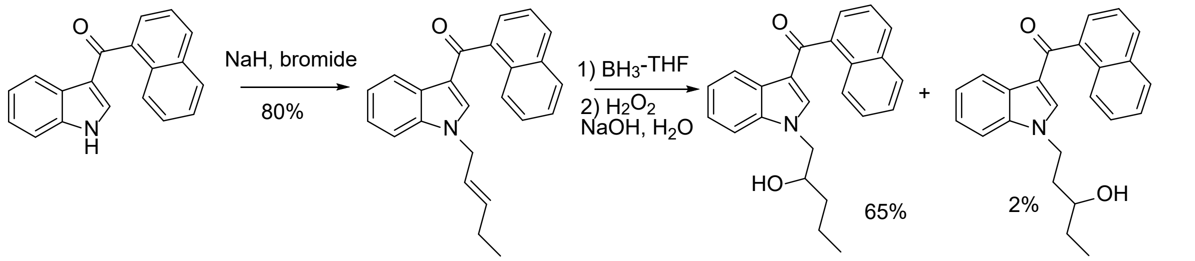

SCM-002 SCM-003

SCM-002: ^{1}H-NMR (300 MHz, $CDCl_3$) δ 8.51 (m, 1 H), 8.18 (m, 1 H), 7.96 (d, 1 H, J = 8.4 Hz), 7.90 (m, 1 H), 7.66 (1 H, dd, J = 7.2, 1.2 Hz), 7.55–7.33 (m, 7 H), 4.19 (m, 1 H), 3.97 (m, 2 H), 1.53–1.39 (m, 4 H), 0.93 (t, 3 H, J = 7.5 Hz). ^{13}C-NMR (75.4 MHz, $CDCl_3$) δ 192.3, 139.1, 139.0, 137.5, 133.9, 130.9, 130.2, 128.4, 127.2, 126.9, 126.5, 126.1, 126.1, 124.7, 123.9, 123.2, 118.0, 110.1, 70.5, 53.2, 36.9, 18.8, 14.1. HRMS (ESI, $[M+H]^+$): Calcd.for $C24H24NO2^+$: 358.1802. Found: 358.1796.

SCM-003: ^{1}H-NMR (300 MHz, $CDCl_3$) δ 8.49 (m, 1 H), 8.19 (m, 1 H), 7.97 (d, 1 H, J = 8.1 Hz), 7.91 (d, 1 H, J = 8.1 Hz), 7.66 (d, 1 H, J = 6.9 Hz), 7.56–7.35 (m, 7 H), 4.27 (m, 2 H), 3.42 (m, 1 H), 1.98 (m, 1 H), 1.81 (m, 1 H), 1.44 (m, 2 H), 0.87 (t, 3 H, J = 7.5 Hz). ^{13}C-NMR (75.4 MHz, $CDCl_3$) δ 192.2, 139.2, 138.4, 133.9, 130.9, 130.2, 128.3, 127.1, 126.8, 126.4, 126.1, 126.0, 124.7, 123.8, 123.1, 123.0, 110.2, 70.1, 43.9, 36.5, 30.8, 9.8. HRMS (ESI, $[M+H]^+$): Calcd.for $C24H24NO2^+$: 358.1802. Found: 358.1799.

(4-hydroxy-1-pentyl-1*H*-indol-3-yl)(naphthalen-1-yl)methanone, JWH-018 4-hydroxyindol (SCM-028)

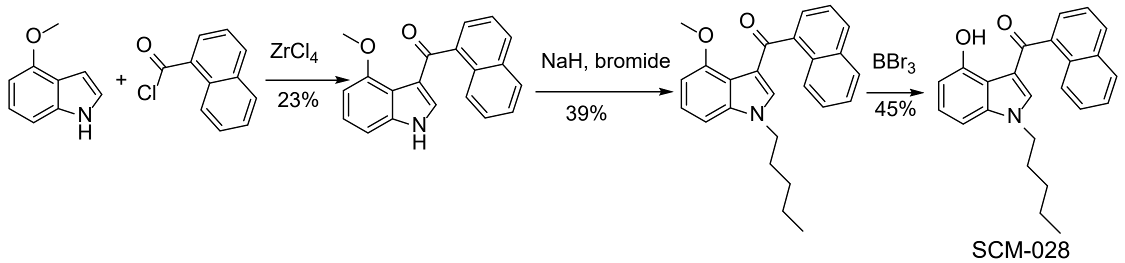

SCM-028

^{1}H-NMR (300 MHz, $CDCl_3$) δ 11.66 (s, 1 H), 8.15 (m, 1 H), 8.01 (d, 1 H, *J* = 8.2 Hz), 7.92 (m, 1 H), 7.66 (dd, 1 H, *J* = 7.1, 1.2 Hz), 7.57–7.51 (m, 3 H), 7.29 (d, 1 H, *J* = 8.2 Hz), 7.24 (m, 1 H), 6.84 (m, 2 H), 4.00 (t, 2 H, *J* = 7.6 Hz), 1.78 (quin, 2 H, *J* = 7.6 Hz), 1.33–1.20 (m, 4 H), 0.85 (t, 3 H, *J* = 7.0 Hz). ^{13}C-NMR (75.4 MHz, $CDCl_3$) δ 193.8, 153.0, 139.5, 137.4, 133.8, 130.9, 130.7, 128.4, 127.2, 126.7, 126.6, 126.2, 125.8, 124.6, 118.5, 116.3, 114.1, 108.6, 101.3, 47.8, 29.3, 29.0, 22.3, 14.0. HRMS (ESI, $[M+H]^+$): Calcd.for $C24H24NO2^+$: 358.1802. Found: 358.1797.

(1-(5-fluoropentyl)-4-hydroxy-1*H*-indol-3-yl)(naphthalen-1-yl)methanone, AM2201 4-hydroxyindol (SCM-029)

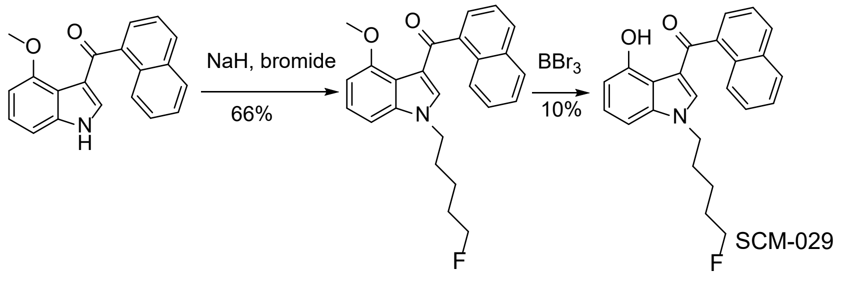

^{1}H-NMR (300 MHz, $CDCl_3$) δ 11.62 (s, 1 H), 8.14 (m, 1 H), 8.00 (d, 1 H, *J* = 8.2 Hz), 7.93 (m, 1 H), 7.66 (dd, 1 H, *J* = 7.0, 1.2 Hz), 7.57–7.49 (m, 3 H), 7.31–7.23 (m, 2 H), 6.84 (d, 2 H, *J* = 8.2 Hz), 4.46 (t, 1 H, *J* = 5.9 Hz), 4.31 (t, 1 H, *J* = 5.9 Hz), 4.03 (t, 2 H, *J* = 7.6 Hz), 1.84 (quin, 2 H, *J* = 7.6 Hz), 1.68 (m, 2 H), 1.42 (m, 2 H). ^{13}C-NMR (75.4 MHz, $CDCl_3$) δ 193.9, 153.1, 139.4, 139.4, 137.3, 133.8, 130.8, 130.8, 128.5, 127.3, 126.7, 126.2, 125.8, 124.6, 118.7, 116.3, 108.6, 101.2, 84.8, 82.6, 47.6, 30.1, 29.8, 29.3, 22.9, 22.8. HRMS (ESI, $[M+H]^+$): Calcd.for C24H23FNO2$^+$: 376.1708. Found: 376.1697.

(1-(5-hydroxypentyl)-1*H*-indazol-3-yl)(naphthalen-1-yl)methanone, THJ-018 5-hydroxypentyl (SCM-014)

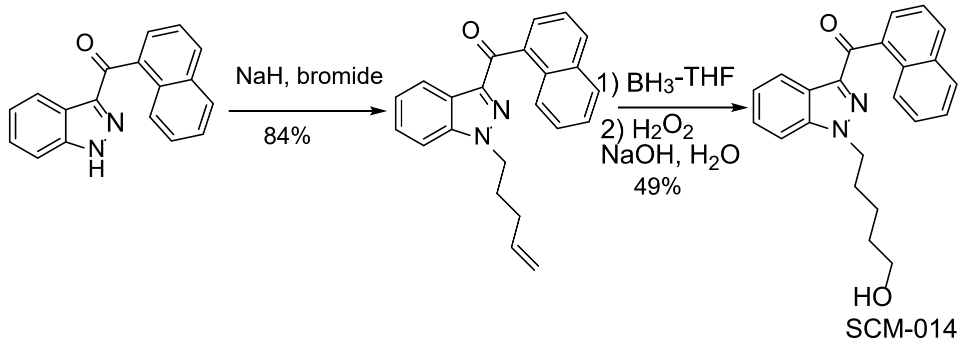

^{1}H-NMR (300 MHz, $CDCl_3$) δ 8.45 (dd, 1 H, *J* = 7.2, 1.2 Hz), 8.33 (m, 1 H), 8.02 (d, 1 H, *J* = 8.1 Hz), 7.96–7.90 (m, 2 H), 7.57 (d, 1 H, *J* = 8.1 Hz), 7.55–7.36 (m, 5 H), 4.43 (t, 2 H, *J* = 7.2 Hz), 3.59 (t, 2 H, *J* = 6.6 Hz), 1.96 (quin, 2 H, *J* = 7.2 Hz), 1.57 (m, 2 H), 1.39 (m, 2 H). ^{13}C-NMR (75.4 MHz, $CDCl_3$) δ 191.8, 142.8, 140.8, 136.5, 134.0,131.6, 131.3, 129.3, 128.5, 127.2, 127.1, 126.3, 125.9, 124.5, 123.9, 123.3, 109.6, 62.6, 49.7, 32.2, 29.6, 23.1. HRMS (ESI, $[M+H]^+$): Calcd.for C23H23N2O2$^+$: 359.1755. Found: 359.1762.

5-(3-(1-naphthoyl)-1*H*-indazol-1-yl)pentanoic acid, THJ-018 pentanoic acid (SCM-036)

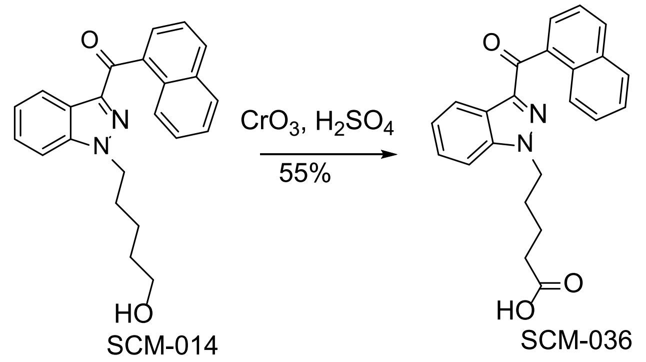

SCM-036. ^{1}H-NMR (300 MHz, $CDCl_3$) δ 8.46 (d, 1 H, *J* = 8.2 Hz), 8.33 (m, 1 H), 8.01 (d, 1 H, *J* = 8.2 Hz), 7.95–7.89 (m, 2 H), 7.57–7.49 (m, 5 H), 7.39 (m, 1 H), 4.44 (t, 2 H, *J* = 7.0 Hz), 2.35 (t, 2 H, *J* = 7.6 Hz), 1.98 (m, 2 H), 1.64 (m, 2 H). ^{13}C-NMR (75.4 MHz, $CDCl_3$) δ 191.7, 177.9, 142.9, 140.8, 136.4, 134.0, 131.6, 131.2, 129.4, 128.5, 127.2, 126.3, 125.9, 124.5, 124.4, 124.0, 123.4, 109.5, 49.3, 33.1, 29.0, 21.9. HRMS (ESI, $[M+H]^+$): Calcd.for $C23H21N2O3^+$: 373.1547. Found: 373.1544.

(1-(2-hydroxypentyl)-1*H*-indazol-3-yl)(naphthalen-1-yl)methanone, THJ-018 2-hydroxypentyl (SCM-020) and (1-(3-hydroxypentyl)-1*H*-indazol-3-yl)(naphthalen-1-yl)methanone, THJ-018 3-hydroxypentyl (26, SCM-021)

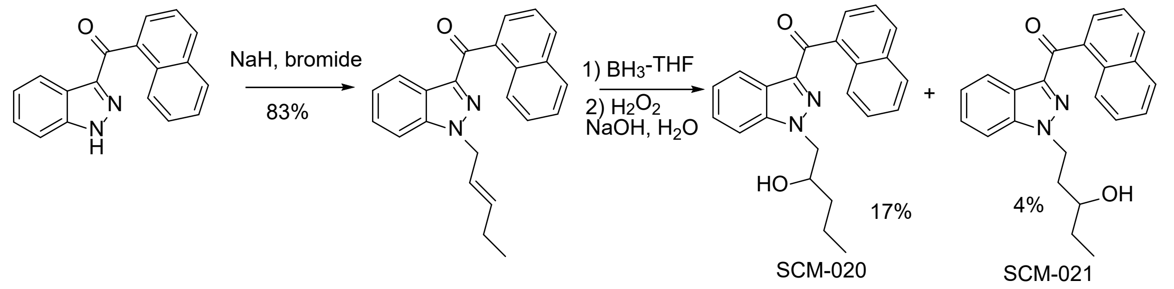

SCM-020. ^{1}H-NMR (300 MHz, $CDCl_3$) δ 8.50 (d, 1 H, *J* = 8.4 Hz), 8.31 (m, 1 H), 8.02 (d, 1 H, *J* = 8.1 Hz), 7.93–7.91 (m, 2 H), 7.58–7.39 (m, 6 H), 4.43 (m, 1 H), 4.29 (m, 1 H), 4.11 (m, 1 H), 2.73 (broad s, 1 H), 1.54– 1.37 (m, 4 H,), 0.91 (t, 3 H, *J* = 6.9 Hz). ^{13}C-NMR (75.4 MHz, CDCl3) δ 191.6, 143.2, 141.7, 136.4, 134.0, 131.7, 131.2, 129.1, 128.6, 127.6, 127.3, 126.4, 125.8, 124.5, 124.2, 124.0, 123.3, 109.8, 70.8, 54.9, 36.7, 18.8, 14.1. HRMS (ESI, $[M+H]^+$): Calcd.for $C23H23N2O2^+$: 359.1755. Found: 359.1763.

SCM-021: ^{1}H-NMR (300 MHz, $CDCl_3$) δ 8.47 (m, 1 H), 8.31 (m, 1 H), 8.02 (d, 1 H, *J* = 8.1 Hz), 7.93–7.90 (m, 2 H), 7.59–7.38 (m, 6 H), 4.59 (m, 2 H), 3.39 (m, 1 H), 2.11 (m, 1 H), 1.89 (m, 2 H), 1.44 (dq, 2 H, *J* = 7.5, 6.6 Hz), 0.87 (t, 3 H, *J* = 7.5 Hz). ^{13}C-NMR (75.4 MHz, $CDCl_3$) δ 191.7, 143.0, 141.0, 136.8, 134.0, 131.6, 131.2, 129.1, 128.5, 127.3, 127.2, 126.3, 125.9, 124.5, 124.3, 124.0, 123.3, 109.7, 70.1, 46.4, 36.4, 30.5, 10.0. HRMS (ESI, $[M+H]^+$): Calcd.for $C23H23N2O2^+$: 359.1755. Found: 359.1766.

General procedures

Organic solvents were concentrated under reduced pressure in a rotary evaporator (LABOROTA 4000, Heidolph) at 30 to 60 °C. THF was distilled over sodium and used immediately. TLC (Sigma Aldrich, Silica gel 60, F254) was carried out using UV-light (CAMAG, 254 nm) and/or PAA-dip (acetic acid: H_2SO_4: p-anisaldehyde 50:1:0.5) for visualization. Silica gel (Merck Grade 9385, high purity grade, pore size: 60 Å, particle size: 0.037–0.063 mm) was used for flash column chromatography. HPLC-MS was performed on a Gilson system (Pump: Gilson gradient pump 322; UV/VIS detector: Gilson 155; MS detector: Thermo Finnigan Surveyor MSQ; Gilson Fraction Collector FC204) with mobile phases (Organic: 90:10 MeCN:H_2O with 10 mM ammonium acetate; Aqueous: 95:5 H_2O:MeCN with 10 mM ammonium acetate), two columns for analytical runs (Waters: X-Bridge C-18 or C-8, 2.5 µm, 50 x 4.6 mm) and one column for preparative analysis (Phenomenex C-18, 5 µm, 100 x 21, 20 mm). LC-QTOF-MS was performed by RMV on an Agilent 6540 system (Jet stream interface, Agilent 1290 Infinity LC instrument, column: ACE Excel 2 C-18-AR, 2 µm, 100 x 2.0 mm). NMR-spectra were recorded on a Varian instrument (^{1}H-NMR: 300 MHz and ^{13}C-NMR: 75.4 MHz).
