# Supplementary material for: Structure-activity relationship of prevalent synthetic cannabinoid metabolites on hCB1 in vitro and in silico dynamics
Source: Acta Pharmacol Sin. 2025 Nov 3;47(3):776–89. doi: 10.1038/s41401-025-01678-5 (PMC12932713; doi:10.1038/s41401-025-01678-5)
Supplement: Supplementary file 3 — Supplementary material B [file 41401_2025_1678_MOESM3_ESM.docx]

# **Supplementary material B**

# ***In vitro* structure relationship activity and molecular dynamic simulation studies of JWH-018, AM-2201, THJ-018, THJ-2201 and their metabolites on the hCB_1_ receptor**

Anna Åstrand^a^, Emiliano Laudadio ^b^, Prince S. Gameli ^c^, Laura Martin^d^, Jeremy Carlier^c^, Francesco P. Busardo^c^, Johan Dahlén^e^, Xiongyu Wu^e^, Peter Konradsson^e^, Svante Vikingsson^a,f,g^, Robert Kronstrand^a,f^ and Henrik Green^a,f^

^a^ Division of Drug Research, Department of Medical and Health Sciences, Faculty of Medicine and Health Sciences, Linköping University, SE 581 85 Linköping, Sweden

^b^ Department of Science and Engineering of Matter, Environment and Urban Planning, Polytechnic University of Marche, Ancona, Italy

^c^ Department of Biomedical Sciences and Public Health, Polytechnic University of Marche, Ancona, Italy

^d^ Institute of Chemistry and Bioanalytics, School of Life Sciences, University of Applied Sciences and Arts Northwestern Switzerland, CH 4132 Muttenz, Switzerland

^e^ Department of Physics, Chemistry and Biology, Linköping University, Linköping, Sweden

^f^ Department of Forensic Genetics and Forensic Toxicology, National Board of Forensic Medicine, SE 587 58 Linköping, Sweden

^g^ Center for Forensic Science Advancement and Application, RTI International, 3040 East Cornwallis Rd., Research Triangle Park, NC, 27709, USA

Corresponding author:

Anna Åstrand

Division of Clinical Chemistry and Pharmacology

Department of Biomedical and Clinical Sciences

Linköping University

SE-581 83 Linköping

Sweden

Mail: anna.astrand@liu.se

***Modeling of CB1 receptor and validation of the Focused Docking approach***

The first step to perform reliable predictions was the modeling of the missing loops of CB1 receptor structure obtained consulting the 6n4b pdb file. For this purpose, the server used was I-tasser, which is a platform devoted to protein structure and function prediction. It enables to generate high-quality model predictions of the 3D structure and biological function of protein molecules from their amino acid sequences. When an aminoacid sequence is analyzed, I-tasser will first work to obtain template proteins of similar folds (or super-secondary structures) from the PDB library as first approach. Then, if it’s necessary, the continuous fragments excised from the PDB templates are reassembled into full-length models using replica-exchange Monte Carlo simulations with the threading unaligned regions built by *ab initio* modeling. If no appropriate template is identified in terms of stability and veracity, I-tasser will build the whole structures by *ab initio* modeling. The low free-energy states are identified through clustering the simulation decoys. In the last step, the fragment assembly simulation is repeated starting from the cluster centroids, where the spatial restrains collected are used to conduct the simulations. In this way, the steric clash are removed to refine the global topology of the cluster centroids. The decoys generated are then clustered again, then the lowest energy structures are selected. The final full-atomic models are obtained building the atomic details through the optimization of the hydrogen-bonding network. The results of the building block of the CB1 missing domains respect to the 6n4b pdb file show the molecular integrity of the structure obtained by preserving the three-dimensionality of the key domains characterizing the binding pocket (Fig. S1A).

Once the reliability of the three-dimensional receptor model was verified, a focused docking investigation was conducted to investigate the binding mode of the crystallized compound. The aim of the investigation was to predict the same binding mode already identified in the crystallographic complex. Only by obtaining this result it is possible to ascertain the reliability of the molecular docking approach. The results obtained show a perfect superposition of the molecule with the original pdb file, indicating the same binding mode and involving the same amino acid pattern (Fig. S1B).

***Rule of MD simulations in the dynamical behavior of compounds***

To find a correlation between the Focused docking clusterization and the predicted binding affinity of each compound, MD simulations have been carried out considering CB1 in complex with 7-OH indole AM-2201, Pentanoic acid JWH-018, and 7-OH indole JWH-018, as representative systems with high, medium, and low population percentage respectively. For this purpose, the physiological conditions together with a cell membrane have been modeled putting the ligand-receptor complexes in their natural environment (Fig. S2A). The binding stability over time for each ligand-receptor complex has been analyzed considering the binding modes of the ligands at the beginning and at the end of the MD simulations (Fig.S2B-D) together with the Root-Mean Square Deviation (RMSD) (Fig. S2E) and Root-Men Square Fluctuation (RMSF) (Fig. S2F).

As result, the 7-OH indole AM-2201, which has high population percentage, maintained the same binding mode detected with the focused docking approach, exhibiting the same RMSD values after 50 ns of starting fluctuations. This implies that the adopted conformation is maintained and refined after the application of the motion equation, demonstrating a solid and stable interaction between compound and receptor over MD time, as also demonstrated by the high population density and very high binding affinity found in the docking approach. The Pentatonic Acid JWH-018, whose binding conformation is medium populated, seemed to slightly move its functional groups in space, which however did not differ much from the initial conformation adopted, given the only slight refinement of the RMSD values after 160 ns. This means that there isn’t an effective interaction between the compound and the receptor, as demonstrated by the relative stability of the conformation adopted and the very low binding affinity found, therefore the molecule does not show any ability to activate the receptor over MD time. Finally, the 7-OH indole JWH-018, for which low population percentage of the binding pose has been detected, showed a more complex stabilization profile, with evident oscillations and a more evident rearrangement in the binding site. This highlights a lower efficacy in the activation of the receptor as well as low population percentage.

The RMSD profile reflects the compound's effect on the fluctuation of the receptor's amino acid residues, as highlighted by the RMSF plots. Indeed, in the presence of 7-OH indole AM-2201, the fluctuation was particularly low, even considering the binding site amino acids. In the complex with Pentatonic Acid JWH-018, the average fluctuation was greater, indicating a slight accommodation of the complex in the binding site. Considering the complex with 7-OH indole JWH-018, this accommodation was more evident, with the binding site amino acids involved in an increased fluctuation. Therefore, a higher stabilization time identified by RMSD is directly correlated with a greater fluctuation of the amino acids induced by compound, and vice versa. Focusing on the chemical modifications, it is possible to note that the 5-OH pentyl metabolites show reduced efficacy but, the 5F-pentyl compound were full agonists. The modifications (eg 5-OH pentyl JWH-018 and AM2201 in comparison to JWH-018) are at the same position but the sizes are different. Indeed, the OH group is larger than the F group even if they have the same number of electrons, and this is due to the higher electronegativity of F respect to OH. F keeps the electron cloud closer to the nucleus (Fig. S3). This also affects the bond length, since the OH group provides a distance between the last C of the chain and the H of the group equal to 1.943 Å in the OH group, while the C-F bond has a total length of 1.400 Å. Another reason of the change in the effect is certainly linked to the dipole of F, which is decidedly higher than the dipole of OH. This depends both on the electronegativity of the C-F bond, but also on the geometry, because C-F bond is linear, while the OH group is not linear, but has a bent geometry, typical of systems with sp3 oxygens. So, the F group is much smaller and much more polarized than OH, influencing the behavior of the compound.
