# Supplementary material for: Structure-activity relationship of prevalent synthetic cannabinoid metabolites on hCB1 in vitro and in silico dynamics
Source: Acta Pharmacol Sin. 2025 Nov 3;47(3):776–89. doi: 10.1038/s41401-025-01678-5 (PMC12932713; doi:10.1038/s41401-025-01678-5)
Supplement: Supplementary file 1 — Supplementary Information [file 41401_2025_1678_MOESM1_ESM.docx]

Dear Editor, Reviewers, and Readers,

As Supplementary Material A, we provide comprehensive background information regarding the in-house synthesis of the metabolites, including the synthetic routes used and the purity obtained.

As Supplementary Material B, we enclose details of the building process, along with a step-by-step approach and validation of the *in silico* method utilized.

We encourage you to use these files to ensure the quality and accuracy of our work.

Yours sincerely, Anna Åstrand & Professor Henrik Green
